# 3D Printing of Mechanical Recycled PA12/CF Composite

**DOI:** 10.3390/polym18202522

**Published:** 2026-10-15

**Authors:** Ionut-Laurentiu Sandu, Felicia Stan, Catalin Fetecau, Andreea-Veronica Botezatu

**Affiliations:** 1 Center of Excellence Polymer Processing, “Dunarea de Jos” University of Galati, 47 Domneasca, 800 008 Galati, Romania; catalin.fetecau@ugal.ro; 2 Department of Chemistry, Physics and Environment, Faculty of Sciences and Environment, “Dunarea de Jos” University of Galati, 111 Domneasca, 800 201 Galati, Romania; andreea.botezatu@ugal.ro

**Keywords:** additive manufacturing, recycling, printability, PA12, carbon fiber

## Abstract

The management of small volumes of polymer and composite waste poses a major challenge for the polymer processing industry because standard recycling plants rely on a continuous, high-volume flow of sorted materials to run. Additive Manufacturing (AM) offers high potential to reprocess small volumes by turning the waste into usable filaments. Although carbon fiber-reinforced polyamide (PA/CF) composites are widely used in engineering applications, comprehensive experimental studies are needed to assess how the end-use properties are retained when recycled PA/CF composites are processed by AM. This study investigates the feasibility of recycling and remanufacturing PA12/CF waste using Fused Filament Fabrication (FFF). The mechanically recycled PA12/CF composite was extruded into filament and 3D printed under different configurations. The physical, chemical, thermal, rheological, microstructural, and mechanical properties of the recycled PA12/CF (rPA12/CF) composite were systematically investigated and compared with those of the virgin counterparts. Experimental results indicated that the recycled PA12/CF composite exhibited very good chemical and thermal stability. However, the mechanical recycling process led to a decrease in the mean CF length, from 0.203 mm in vPA12/CF to 0.147 mm in rPA12/CF, as well as a reduction in melt shear viscosity. The mechanical properties of the rPA12/CF composite depend on the printing configuration. The rPA12/CF samples printed at 0° orientation retained at least 90% of the elastic modulus or bending modulus values of the vPA12/CF counterpart, while the tensile strength and ductility increased by 10%. This study confirms the potential of FFF technology to reprocess small volumes of recycled composite waste into specialized parts where the balance between the cost and end-use properties justifies the use of recycled materials.

## 1. Introduction

Additive manufacturing (AM) is a flexible, cost-effective, feasible, and sustainable solution for manufacturing parts from thermoplastic polymers [1,2,3]. Comprehensive studies [4,5,6,7,8,9,10] have reviewed the major advantages, technological evolution, current applications, and prospects of AM technologies. These studies highlight their growing impact across industrial sectors and the global market for 3D printing products and services. Continuous advances in AM equipment, processing technologies, and the development of novel polymer composites with enhanced and multifunctional properties have accelerated the adoption of these technologies in increasingly demanding engineering applications. In the context of the transition toward a circular economy and sustainable manufacturing, the use of recycled polymers and polymer composites in AM continues to expand. Therefore, a true understanding of the changes induced by recycling is essential to ensure reliable performance, extend material service life, and enable their sustainable reintegration into circular manufacturing systems. One of the most widely used material extrusion-based AM technologies is Fused Deposition Modeling (FDM), also known as Fused Filament Fabrication (FFF). This technology enables the layer-by-layer fabrication of thermoplastic parts from a digital CAD model [11].

Recent advances in materials and processing technologies [12,13,14,15] have expanded the range of feedstocks available for AM, including pellets, powders, and continuous filaments. These feedstocks enable the processing of polymers, spanning from general-purpose materials, such as PLA and PETG for prototyping, to engineering thermoplastics such as ABS, polyamides, POM, PC, and PBT [16,17,18,19,20,21]. High-performance polymers, including PEEK and PEKK are also increasingly used for demanding functional applications [17,18,22,23]. Among these materials, polyamide 12 (PA12) is highly valued for its excellent mechanical and chemical resistance, making it a suitable candidate for the fabrication of functional components using FDM/FFF [24]. To enhance the mechanical properties of the 3D-printed parts, these polymers can be reinforced with fibers or particles such as carbon, carbon black, graphite, CNTs, glass, aramid, as well as calcium carbonate, basalt, clay, or silica [25,26,27,28]. Another benefit of adding fillers is the introduction of additional functionalities, such as electrical or thermal conductivity [29,30].

Mechanical recycling methods provide a more economical and simpler alternative to more complex chemical recycling processes [31,32]. Mechanical recycling converts thermoplastic waste and scraps into functional secondary raw materials, such as flakes and pellets, thereby supporting circular economy principles [33,34]. Compared with the production of virgin polymers, mechanical recycling can reduce energy consumption by avoiding several energy-intensive stages associated with the extraction and processing of raw materials, monomer production, and polymerization [35]. The resulting recycled materials can subsequently be reintroduced into conventional polymer-processing operations, such as extrusion and injection molding, using processing equipment and workflows similar to those employed for virgin thermoplastics [34,35].

However, mechanically recycled plastics and composites may degrade during repeated processing. For example, polymer chains may undergo shortening [36,37,38,39,40,41] during each recycling cycle, resulting in reduced mechanical strength. Fiber fragmentation may also occur in fiber-reinforced composites [42,43,44]. These degradation mechanisms can lead to a gradual deterioration in the mechanical properties of the material [45,46,47,48,49]. Depending on the polymer and processing conditions, mechanically recycled plastics may undergo only a limited number of recycling cycles before significant losses in structural integrity and performance occur. Consequently, the addition of virgin material may still be required. Furthermore, mechanical recycling requires rigorous sorting and washing procedures to ensure adequate material quality and consistency.

In recent years, FDM/FFF technology has been successfully used to reprocess small volumes of polymers and polymer composites (custom-made) waste [10,36,50,51,52], including waste generated during 3D-printing and other industrial processes, such as injection molding and extrusion. However, despite the economic and environmental benefits, the main limitation of using recycled materials in AM is the potential loss of mechanical performance. Previous studies have reported that 3D-printed parts made from recycled materials generally exhibit lower mechanical performance than those made from virgin materials [10,36,50,53,54,55,56,57,58]. This reduction in mechanical performance can be attributed to several degradation mechanisms and processing-related factors, including thermo-oxidative degradation processes [37,38,39,40,41], chain fragmentation, scission or branching [36,37,38,40,41,59], cross-linking [36,37,39,40,41], loss of molecular weight [36,37,38,39,40,41], prolonged residence time and associated polymer degradation [40,41], and changes in melt viscosity [36,37,38,39,41,59].

Several experimental studies on the recycling and reprocessing of thermoplastic composites using FFF have reported promising retention of functional and mechanical properties. These studies also highlight the potential economic and environmental benefits of material reuse and waste reduction. Fico [60] has shown that 3D printing can be used as a low-cost method for reprocessing recycled composites, offering environmental and economic benefits, although 3D-printed objects produced from 100% recycled PLA/ceramic filament exhibit reduced mechanical performance. Giani [61] investigated the effects of recycled carbon fiber (CF) content and deposition angle on the thermo-mechanical properties of a 3D-printed PLA composite. The experiments demonstrated that CF alignment relative to the applied stress direction resulted in strong anisotropy in the mechanical properties. Moreover, adding up to 10 wt.% CF reduced thermal expansion by up to 85% compared to pure PLA and a substantial increase in the storage modulus to 7.1 GPa. Tian [62] employed an innovative FFF-based method to maintain or even enhance the mechanical performance of the printed parts. This method involved the use of a continuous CF and a thermoplastic matrix for 3D-printing parts after a reverse trajectory, allowing for 100% recycling of the continuous fiber. Thus, the 3D-printed samples exhibited a 25% higher bending strength than the original ones. The recovery rates for the continuous CF and thermoplastic matrix were 100 and 73%, respectively. Ateeq [63] investigated the fabrication of PA12 composite filaments filled with recycled CF (rCF) recovered from end-of-life aircraft. The resulting composite filaments were subsequently used to manufacture parts by FFF. The study showed that the filament extrusion can produce rCF-reinforced PA12 filaments with a dimensional tolerance of ± 0.05 mm. These filaments were successfully processed by FFF to manufacture composite parts with improved flexural performance. The addition of rCF (10 and 15 wt.%) consistently improved the mechanical properties of 3D-printed parts compared with neat PA12, regardless of infill density. Zanelli [64] studied the recycling of short CF-reinforced PA6 composite using FFF. The study showed that grinding and re-melting affected the rheological properties of the recycled PA6 composite by reducing its molecular weight and complex viscosity. However, the thermal properties of both virgin and recycled composites did not show a significant change, nor did the crystallinity degree. The recycled 3D-printed samples were also found to be more brittle than the virgin samples, regardless of the printing orientation. Bandinelli [65] investigated the feasibility of mechanically recycling and manufacturing PA12 reinforced with 10 wt.% short CF using 3D-printing techniques, namely Fused Granular Fabrication (FGF) and (FFF). The study showed that the recycled PA12/CF composite did not exhibit substantial differences in chemical and physical properties compared with the virgin composite. However, notable differences in the microstructure and mechanical properties of the 3D-printed samples were observed between FGF and FFF technologies. The recycled tensile samples were 24 and 6% denser than the virgin FFF and FGF, respectively, resulting in increases in stiffness and ultimate strength of up to 43 and 20%, respectively. In contrast, the higher density of the recycled samples was associated with a 26% reduction in energy absorption compared with the virgin samples. Other studies [66,67] have also demonstrated that AM technologies such as Selective Laser Sintering (SLS) or Multi Jet Fusion (MJF) can be successfully employed to reprocess thermoplastic polymers, with or without fibers or particles reinforcement, thereby supporting their integration into the circular economy practices.

For high-performance polymers such as PA12 reinforced with CF, the choice of recycling process requires a balance between cost and material performance. In this context, combining mechanical recycling with 3D printing represents a viable approach for recovering and reusing PA12/CF waste, while reducing material consumption and supporting more sustainable manufacturing practices. Studies [21,24,68] have shown that the chemical and physical properties of the PA12 polymer undergo minimal degradation during the first few re-melting cycles.

Despite these promising results, studies investigating the mechanical recycling and reprocessing of PA12 reinforced with CF through AM remain relatively limited, particularly regarding the comprehensive assessment of the effects of recycling on material properties and printability. Moreover, the combined thermal, chemical, morphological, rheological, and mechanical changes induced by recycling and AM reprocessing of PA12/CF composites have not yet been sufficiently investigated.

This study therefore investigates the recycling and FFF reprocessing of a PA12/CF composite. PA12 reinforced with CF was selected because of its extensive use in additively manufactured components, excellent mechanical and thermal properties, and high specific stiffness. These characteristics make PA12/CF composites attractive candidates for mechanical recycling while retaining the functional performance required for lightweight components. The novelty of the present study consists of a systematic investigation of a mechanically recycled PA12/CF composite containing 20 wt.% CF using FFF technology. Thermal, chemical, morphological, rheological, and mechanical analyses were combined to comprehensively assess the effects of recycling on material properties and printability. The study also evaluates the feasibility of reusing the recycled PA12/CF composite through FFF and provides further insight into the effects of mechanical recycling and reprocessing on its material performance.

## 2. Materials and Methods

### 2.1. Raw Composite, Recycling and Filament Fabrication (Extrusion)

In this study, a standard 1.75 ± 0.05 mm diameter composite filament based on polyamide 12 (PA12) and containing 20 wt.% carbon fiber (CF) (Fiber Force NYLFORCE CF, batch number CT0963, Fiber Force, Treviso, Italy [69]) was used as the virgin PA12/CF composite (vPA12/CF). According to the manufacturer, the main characteristics of the vPA12/CF composite are as follows: density of 1 g/cm^3^, melting point 180 °C, tensile modulus of 6 GPa, tensile strength of 0.1 GPa, impact strength of 60 kJ/m^2^, recommended printing temperature of 250–265 °C, print speed of 40 mm/s and bed temperature of 60–70 °C [69].

**Figure 1:**
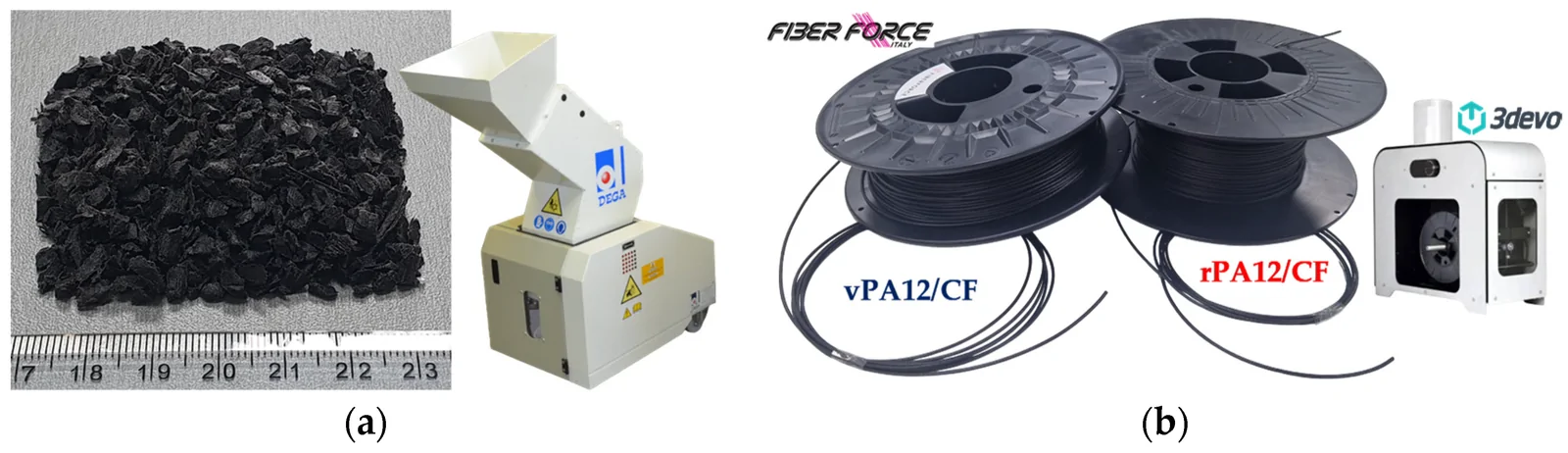
PA12/CF composite (**a**) recycled flakes; (**b**) rPA12/CF 1.75 mm filament.

The recycled PA12/CF composite (rPA12/CF) was obtained from 3D-printed scrap parts, including rafts, brims or skirts, as well as 3D-printed samples remaining after different characterization tests. The vPA12/CF was first ground into 2 to 5 mm flakes (see Figure 1a) using a roller shredder (GS 17/22, Dega Plastics, Brescia, Italy). The resulting flakes were then dried at 90 °C for 12 h in a vacuum of −0.95 bar and directly extruded into 1.75 mm diameter filaments (see Figure 1b) using a desktop filament maker (NEXT 1.0, 3Devo, Utrecht, The Netherlands). The rPA12/CF filament was extruded at a melt temperature of 185 °C, screw speed of 7 rpm, and extruder speed of 700 rpm. The speed tuning was set to automatic (≈1700 rpm), while the fan speed was maintained between 15 and 20%. The vPA12/CF waste was stored in a clean environment; therefore, the no washing step was applied before recycling.

### 2.2. Fused Filament Fabrication-FFF

**Figure 2:**
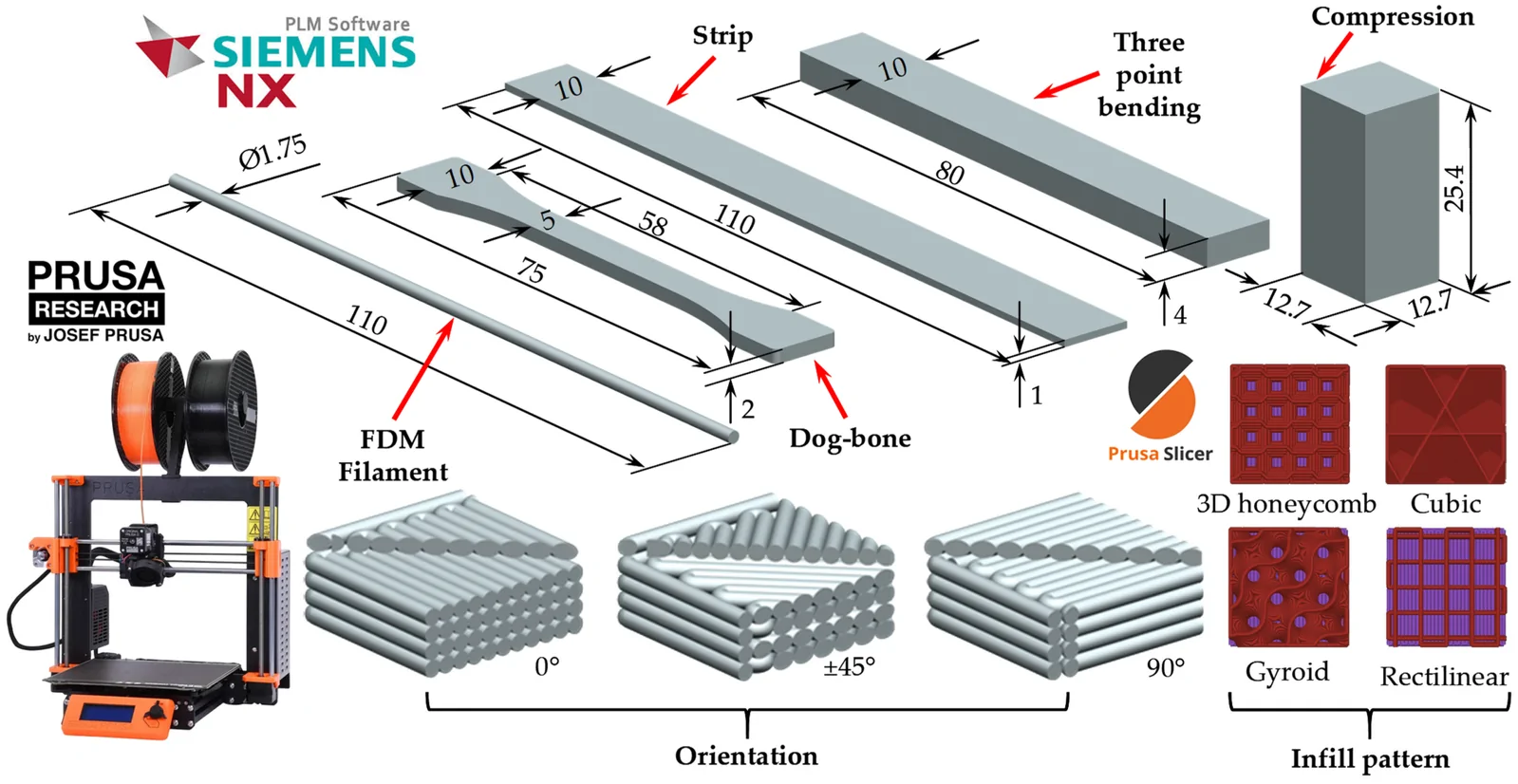
Geometry, orientation, and infill pattern for 3D-printed samples.

FFF of the vPA12/CF and rPA12/CF composite filaments (see Figure 2) was performed using a 3D printer (Original Prusa i3 MK3S+, Prusa Research, Prague, Czech Republic). All samples were designed using Siemens NX 7.5 PLM software, with dimensions specified according to ISO 527 [70] for tensile (dog-bone), ISO 178 [71] for three-point bending, and ISO 604 [72] for compression samples. The geometry and dimensions of the samples, including the filament, are presented in Figure 2. The PA12/CF compression samples were 3D-printed without perimeters (outer shells) to allow direct observation of the cellular deformation and failure modes under compressive loading and to investigate the performance of sandwich panels and structures.

The samples were sliced for 3D-printing in PrusaSlicer 2.7.4 using Marlin 1 as the G-code firmware. The 3D-printing parameters are presented in Table 1. Prior to printing, the filaments were dried for 12 h at 90 °C in a vacuum oven (EV-50, Raypa, Barcelona, Spain) at a vacuum pressure of −0.95 bar. The nozzle temperature was set based on the filament manufacturer specifications and was correlated with the DSC and MFI results. At least ten samples were 3D-printed for each configuration and stored under laboratory conditions before testing. To investigate the effects of orientation and infill pattern on the mechanical behavior of 3D-printed composites under tensile, three-point bending, and compression, several configurations were considered, as shown in Figure 2.

The printability of the PA12/CF composite was characterized using the uniformity index (UI) for linear dimensions and the relative error in the weight of the 3D-printed parts. The uniformity index is a critical metric for 3D-printing that reflects the quality of 3D-printed parts [73,74,75,76]. In this study, the length, width, and thickness/height of the 3D-printed parts were measured 24 h after 3D-printing. The UI was calculated as a percentage by dividing the experimental measured dimension of the 3D-printed part by the corresponding theoretical nominal dimension shown in Figure 2 [73,74,75,76].

### 2.3. Characterization of the PA12/CF Composite

#### 2.3.1. Solid Density

The solid density of the PA12/CF composite was measured according to the ISO 1183-1 standard [77] and the Archimedes principle. Filament and 3D-printed samples of the PA12/CF composite were weighed in ethanol and air using an analytical balance (AB204-S/FACT, Mettler Toledo, Columbus, OH, USA) equipped with a density kit. The average solid density of the PA12/CF composite was calculated using ten independent determinations.

#### 2.3.2. Ash Content and CFs Length

The ash content can be used to monitor changes in the composite mass associated with the mechanical recycling process. The analysis can be reliable only if the oxidation temperature of the filler exceeds the ashing (calcinations) temperature of the polymer matrix. Thus, the ash content analysis of the vPA12/CF and rPA12/CF composites was performed according to ASTM D5630 [78] using a muffle furnace (LE1/11, Nabertherm GmbH, Bremen, Germany), porcelain crucibles, and a desiccator filled with calcium chloride desiccant. To assess whether the recycling process affected the CF content within the PA12 matrix, porcelain crucibles containing approximately 2 g of vPA12/CF and rPA12/CF were heated for 30 min in the preheated muffle furnace at 500 °C. This temperature was selected based on the DSC curves to completely oxidize the PA12 polymer matrix [79,80] without degrading the CF [81,82]. The PA12/CF composite began to oxidize at temperatures above 440 °C and exhibited a peak around 465–475 °C. Subsequently, the crucibles were cooled to room temperature (approx. 40 min) in a desiccator and weighed using an analytical balance (AB204-S/FACT, Mettler Toledo, Columbus, OH, USA). The ashing process was repeated three times, with the ashing period reduced to 5 min. The ash content (degree) was calculated using Equation (1) [78]

(1) $$ash\;mass=\frac{\left(W_{3}-W_{1}\right)}{\left(W_{2}-W_{1}\right)}\cdot 100\;(\%),$$

in which *W*_1_ is the weight of the empty dry crucible, *W*_2_ is the weight of the crucible filled with the composite, and *W*_3_ is the weight of the crucible with the remaining ash after the test.

The length of the CFs in both virgin and recycled PA12/CF filaments was analyzed following the ash content procedure by measuring the individual fiber lengths after the PA matrix was burned off. The isolated fibers were spread on a flat surface and observed, and about 400 fibers were measured using an optical Stereo Microscope System (SZX10, Olympus, Tokyo, Japan) equipped with image analysis software (QuickPHOTO MICRO 3.2, PROMICRA, Praga, Czech Republic).

#### 2.3.3. Fourier Transform Infrared Spectrometry

The thermo-oxidative degradation induced by the mechanical recycling of the PA12/CF composite was monitored by Fourier transform infrared (FT-IR) spectroscopy. Measurements were performed using a spectrometer (Nicolet™ iS50 FT-IR, Thermo Fisher Scientific Inc., Waltham, MA, USA) equipped with a built-in ATR accessory, a DTGS detector, and a KBr beam splitter. Spectra were acquired by co-adding 32 scans in the 4000–500 cm^−1^ region with a spectral resolution of 4 cm^−1^.

#### 2.3.4. Differential Scanning Calorimetry

The effect of the mechanical recycling process on the thermal behavior of the PA12/CF composite was investigated using a differential scanning calorimetry (DSC) instrument (DSC 3 STAR^e^ System, Mettler Toledo, Columbus, OH, USA). Virgin and recycled samples, each weighing about 5 mg and taken from the filaments and 3D-printed samples, were sealed in a 40 μL aluminum pan and tested at a scanning rate of 10 °C/min between 25 and 300 °C for two endothermic scans and one exothermic scan. The first heating scan was used to erase the previous thermal history, while the cooling scan was used to determine the glass transition temperature (T_g_) and crystallization temperature (T_c_). The second heating scan was used to determine the melting temperature (T_m_), melting enthalpy (ΔH_m_), and degree of crystallinity (χ_c_). The crystalline content (χ_c_) was calculated using Equation (2) [83]

(2) $$\chi_{c}=\frac{1}{1-wt.\%}\cdot \frac{\Delta H_{m}}{\Delta H_{m}^{0}}\cdot 100\;(\%),$$

in which, $\Delta H_{m}^{0}$ is the melting enthalpy of 100% crystalline polymer, and wt.% is the CF weight fraction. The melting enthalpy of 100% PA12 was taken as 209.3 J/g [84].

#### 2.3.5. Rheological Characterization

To evaluate the effect of the melt extrusion temperature on the flow behavior [85,86] of vPA12/CF and rPA12/CF composites, melt flow index (MFI) measurements were carried out based on the ISO 1133 test method [87]. Measurements were performed using an extrusion plastometer (Melt Flow Quick Index 7021-7022, Instron Corp., Norwood, MA, USA) at three extrusion temperatures (240, 250, and 260 °C) under a load of 2.16 kg. Five MFI measurements were performed for each of the vPA12/CF and rPA12/CF composites to assess the variability of the results. Before all MFI experiments, moisture was removed from the PA12/CF composite using a vacuum oven (EV-50, Raypa, Barcelona, Spain). Therefore, the vPA12/CF and rPA12/CF pellets were dried at 90 °C for 12 h under a vacuum of −0.95 bar.

Capillary rheology tests were performed on both virgin and recycled PA12/CF filaments at 250 °C using a capillary rheometer (RG75, Göttfert, Buchen, Germany) equipped with a capillary die with a length-to-diameter ratio (L/D) of 10/1. The PA12/CF filaments were cut into pellets up to 5 mm in length and vacuum-dried at 90 °C under a vacuum of −0.95 bar for 12 h in an oven (EV-50, Raypa, Barcelona, Spain). The initial melting time of the PA12/CF pellets was set to 600 s, and four shear rates relevant to the 3D-printing process, ranging from 100 to 1000 s^−1^, were selected.

#### 2.3.6. Morphological Analysis

The influence of the mechanical recycling process on the dispersion of CF within the PA12 matrix and the morphology of the 3D-printed composites was investigated using a scanning electron microscope (SEM) (Quanta 200, FEI, Hillsboro, OR, USA) operated at 15 kV. Virgin and recycled dog-bone samples printed at 0, ± 45, and 90° orientations were selected and cryo-fractured in liquid nitrogen after the cross-section. A 5–10 nm layer of gold-based alloy was sputtered onto the cross-sectional surface of the samples before SEM imaging.

#### 2.3.7. Mechanical Characterization

All mechanical tests, including tensile, three-point bending, and compression tests, were performed using a universal testing machine (M350-5AT, Testometric, Rochdale, UK). The tensile tests were performed at a crosshead speed of 1 mm/min with a gauge length of 58 mm for the filaments, at a crosshead speed of 5 mm/min with a gauge length of 58 mm for dog-bone samples, and a crosshead speed of 1 mm/min with a gauge length of 60 mm for the rectangular strips. The three-point bending tests were carried out at a crosshead speed of 1 mm/min and a support span of 40 mm, while the compression tests were performed at a crosshead speed of 1 mm/min without any preload. To determine the mean and standard deviation of the mechanical properties, at least five independent samples were tested for each configuration, in accordance with the ISO 527 [70], ISO 178 [71], and ISO 604 [72] standards, respectively.

## 3. Results and Discussion

### 3.1. Physical Properties

#### 3.1.1. Solid Density

To investigate the effect of the mechanical recycling process and reprocessing through AM on the physical properties of the PA12/CF composite, solid density was evaluated, as changes in density may reflect alterations in the polymer molecular structure and crystalline morphology associated with thermo-mechanical degradation, including chain branching, chain scission, and crosslinking [40,88,89,90]. In addition, solid density can provide an indication of changes in the CF content resulting from mechanical recycling [57,91].

Table 2 shows the solid density results for the vPA12/CF and rPA12/CF composites. The density of the vPA12/CF filament is about 1.01 g/cm^3^ and remains unchanged after the mechanical recycling and extrusion process. Similarly, no significant change in solid density was observed between the virgin and recycled 3D-printed PA12/CF composites. However, the solid density of the 3D-printed samples was approximately 7% lower than that of the corresponding filaments. This reduction may be attributed to the presence of air gaps (micro-air bubbles) and internal voids generated during the FFF process, which are commonly associated with the layer-by-layer deposition of material, as reported by other researchers [92,93,94].

#### 3.1.2. Ash Content and CFs Length

Table 3 shows the ash content of the PA12/CF composite after successive flame cycles at 500 °C. The vPA12/CF composite exhibits a higher ash content (6.44–6.32 wt.%) than the rPA12/CF sample (5.86–5.77 wt.%), suggesting that the recycling process may have caused CF loss or degradation. After the fourth ashing cycle, the CF/ash wt.% from the PA12/CF composite stabilizes at 6.32 wt.% for the vPA12/CF and 5.7 wt.% for the rPA12/CF. However, the difference between the virgin and recycled PA12/CF is relatively small (less than 9%), indicating that the composite has good structural stability during the recycling process. A single flame of 30 min at 500 °C is enough to determine the ash (unoxidized CF) wt.% from the PA12/CF composite; both composites react similarly to repeated flames, and the cumulative decrease in ash content during the additional flames is minimal (up to ~0.12 for vPA12/CF and ~0.09 wt.% for vPA12/CF).

**Figure 3:**
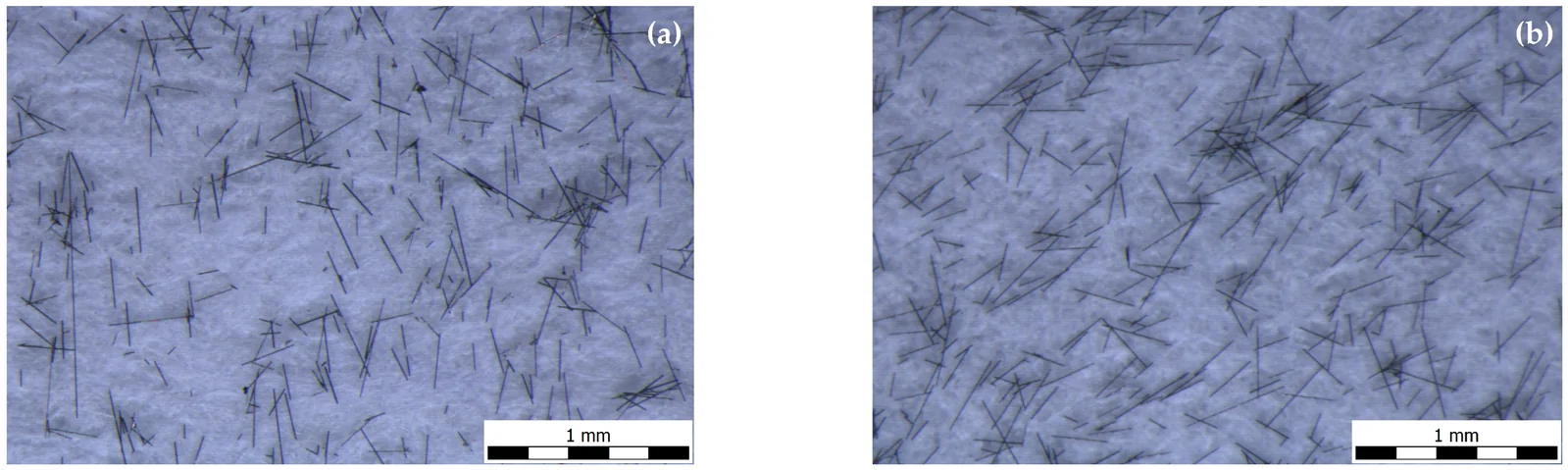
Optical micrographs of the individual CFs from virgin (**a**) and recycled (**b**) PA12/CF filament.

Figure 3 shows representative optical micrographs of the CFs from the vPA12/CF filament (Figure 3a) and rPA12/CF filament (Figure 3b), where it is clearly observed that they possess different length. Since fiber length distributions are skewed and asymmetrical, the average and standard deviation were not sufficient to describe the distribution of the CF lengths [95,96]. Therefore, quartiles were used for comparative analysis between the CF lengths from the vPA12/CF and rPA12/CF, and results are presented in Table S2.

The recycling process (e.g., mechanical recycling followed by filament extrusion) reduces the fiber length metrics significantly (Table S2). The number-average length drops by about 28% (from 0.204 mm in vPA12/CF to 0.147 mm in vPA12/CF), and the weight-average length decreases by 25%. The maximum fiber length drops from 0.510 mm in the virgin filament to 0.430 mm in the recycled filament. The 75th percentile (Q_3_) indicates the domain where the top 25% longest fibers reside. In the virgin filament (vPA12/CF), about 25% of all fibers are longer than 0.263 mm, while in the recycled filament (rPA12/CF), the threshold decreases to 0.188 mm. The interquartile range (IQR) contains the middle 50% of the CF data. The CFs from the vPA12/CF features a broad bulk distribution spanning from 0.120 mm to 0.263 mm. On the other hand, the CF from the rPA12/CF features a narrow distribution of 0.080 mm to 0.188 mm, indicating that the recycling shear forces trim the CF with higher lengths (L_max_ decreases from 0.510 to 0.430 mm, Table S2) and create a shorter, more uniform distribution. Finally, the 25th percentile (Q_1_) shows that 25% of the CF lengths of the rPA12/CF have been crushed into fibers under 0.080 mm, compared to 0.120 mm in the vPA12/CF filament. These fine CF fragments may increase the melt viscosity of the polyamide matrix during 3D printing. Moreover, since the mechanical stress transfer relies on the longest fibers, shorter CF (fine fragments) may have a negative effect on the tensile strength of the recycled filament.

### 3.2. FT-IR Spectroscopy

**Figure 4:**
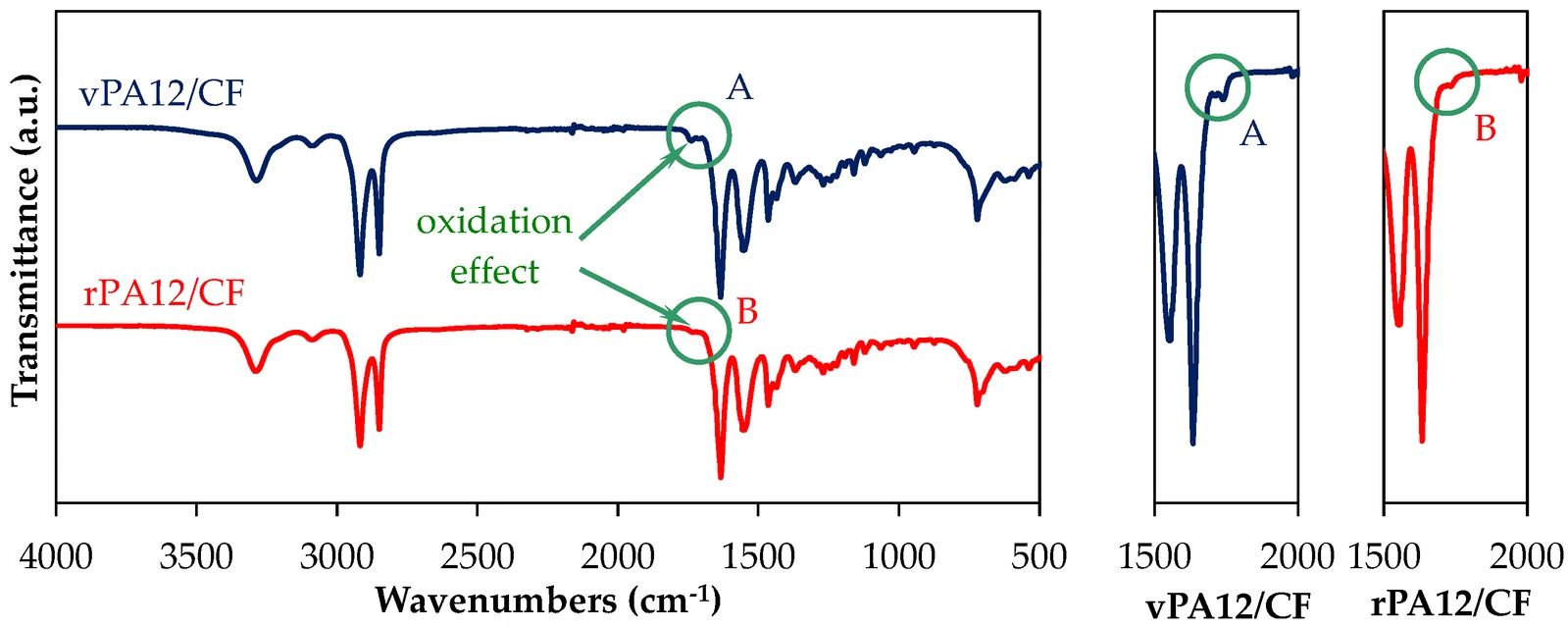
FT-IR spectroscopy of PA12/CF composite.

Figure 4 shows the FT-IR spectra of the PA12/CF composite. The results indicate high chemical stability of the rPA12/CF, with spectra almost identical to those of the vPA12/CF. The main PA12 functional groups are clearly visible in both samples: ~3290 cm^−1^ N-H stretching vibrations, ~2850 and ~2920 cm^−1^ C-H stretching (from the hydrocarbon chain), ~1635 cm^−1^ (Amide I) primarily assigned to the C=O stretching vibration, and ~1555 cm^−1^ (Amide II) relates to N-H bending and C-N stretching [97,98,99]. For rPA12/CF, an oxidation effect is observed at 1738.38 cm^−1^, represented by the formation of free carboxylic groups of ketones, aldehydes, or aliphatic esters that arise during the thermal processing, mechanical shearing, or aging processes that the recycled composite undergoes [99,100,101]. The absence of a much stronger peak than the one observed at around 1635 cm^−1^ (formation of additional carbonyl groups through oxidation) suggests that the mechanical recycling process was well controlled with minimal oxidation. Similar results were observed in the literature for recycled PLA reinforced with CF [102].

### 3.3. Thermal Properties

**Figure 5:**
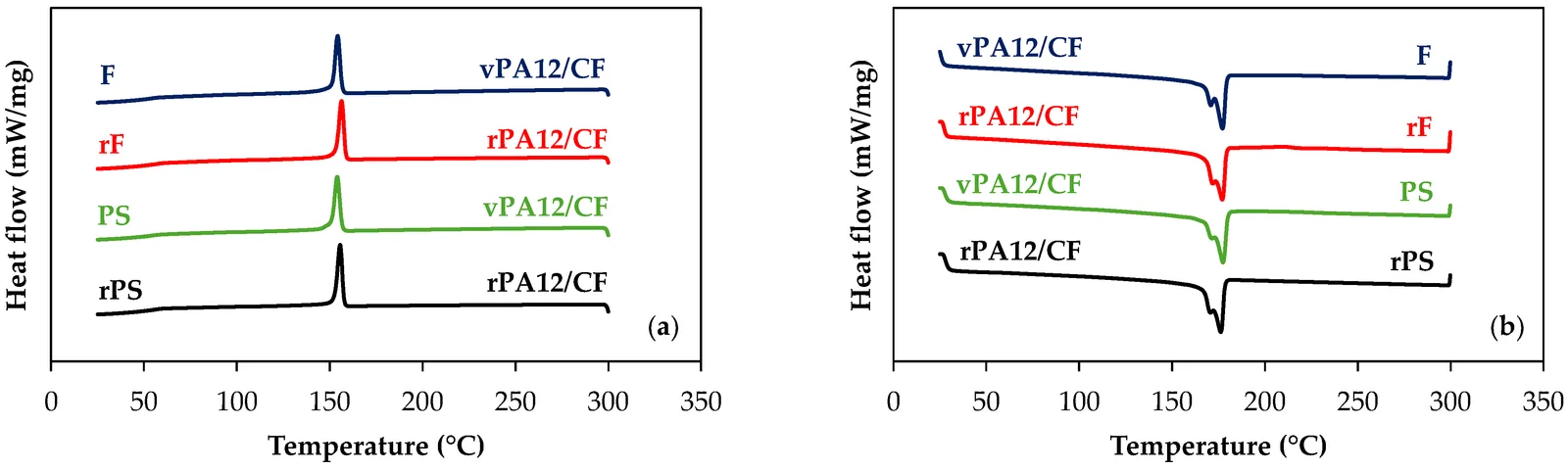
DSC thermograms of the PA12/CF composite: (**a**) first cooling, (**b**) second heating.

Figure 5 shows the influence of mechanical recycling on the DSC thermograms of the PA12/CF composite. The thermal properties extracted according to ISO 11357 [103,104] from the first cooling and second heating scans are summarized in Table 4. According to the results, both vPA12/CF and rPA12/CF composites exhibited similar behavior with few differences in the T_c_ and X_c_. The increase in the T_c_ of the recycled composite (by about 2 °C) is associated with CF shortening and possibly with chain scission induced during recycling. Similar results have been reported in the literature [36,68]. Due to chain scission, long chains break into shorter chains that are easier to move and rearrange, causing an increase in molecular mobility. Thus, for the recycled polymers, crystallization during cooling begins earlier at higher temperatures [36,68].

The X_c_ increased from about 25% for the virgin filament to about 29% for the recycled filament. This increase may be attributed to the combined effects of the thermal history associated with mechanical recycling and reprocessing that shorten the CFs. As shown in Table S2, for the rPA12/CF filament, about 25% of fibers have been crushed into micro-particles under 0.08 mm, compared to 0.12 mm in the vPA12/CF filament. These small fibers increase the number of nucleation sites that promote the nucleation of more crystals. The X_c_ of the rPA12/CF 3D-printed parts slightly decreased to about 27%, corresponding to a reduction of approximately 5% relative to the rPA12/CF filament. This may be associated with severe cooling conditions during 3D printing that hinder the growth of crystals. Nevertheless, the X_c_ of the rPA12/CF 3D-printed rPA12/CF parts is approximately 3% lower than that of the 3D-printed vPA12/CF parts, indicating that the recycling history marginally suppresses the crystallization capability of the PA12 matrix during the 3D printing process.

The DSC analysis indicated that the recycling process has a negligible effect on the T_g_ and T_m_, preserving the core thermal stability of the composite without degrading structural transition phases. Both recycled and virgin 3D-printed PA12/CF samples exhibit a T_g_ of 53 °C and a T_m_ of 176 °C, with a crystalline polymorphism induced by CFs (T_shoulder_) at 170 °C.

### 3.4. Rheological Properties

**Figure 6:**
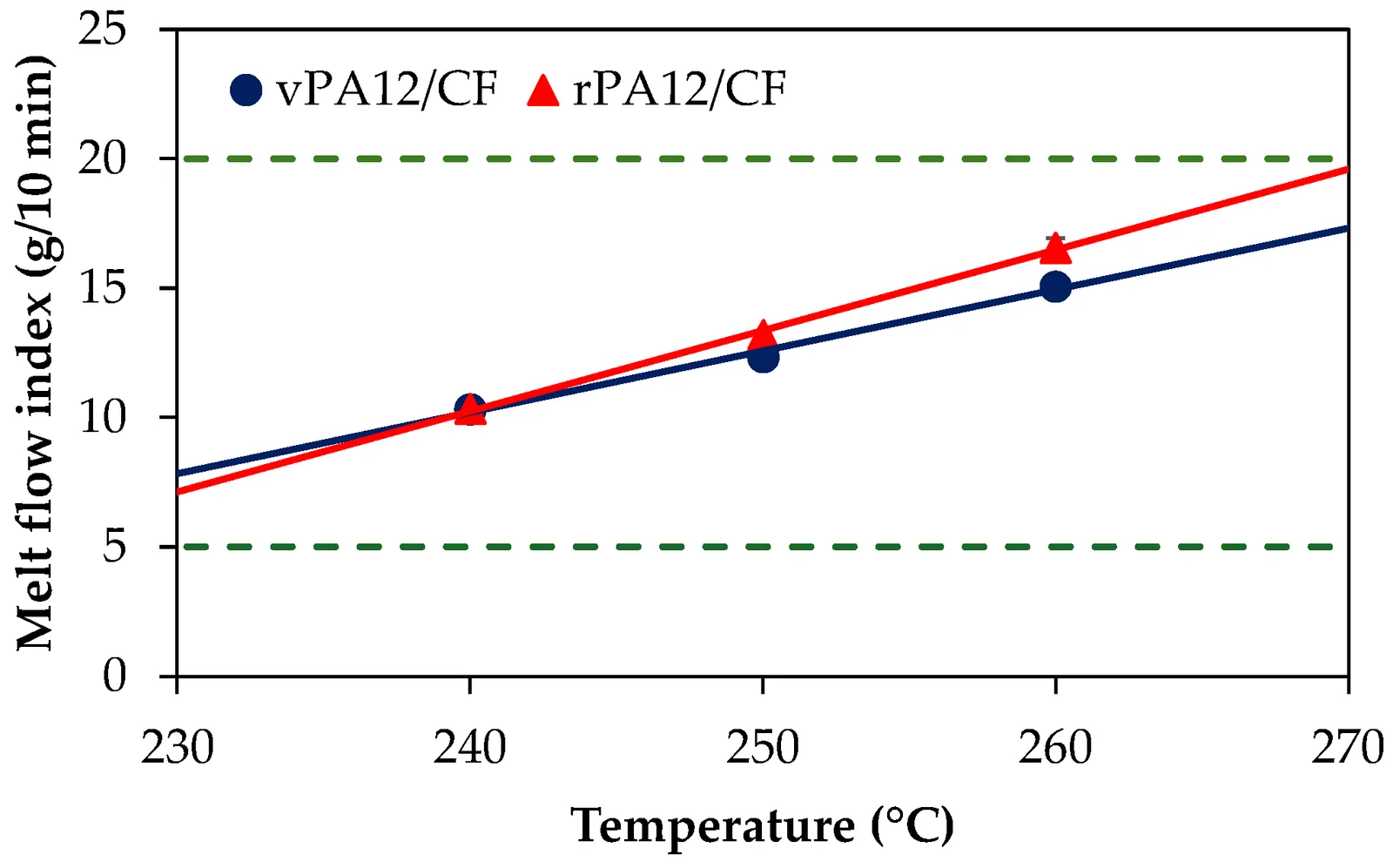
Effect of the mechanical recycling process and extrusion temperature on the PA12/CF composite MFI.

Figure 6 presents the effect of the recycling and extrusion temperature on the MFI of the PA12/CF composite. It is observed that the rPA12/CF exhibits slightly higher MFI values than the vPA12/CF, particularly at 250 and 260 °C. At 240 °C, both vPA12/CF and rPA12/CF exhibit an MFI close to 10 g/10 min. As the temperature increases from 240 to 260 °C, the MFI progressively increases for both composites, reaching 12 and 13 g/10 min at 250 °C and approximately 15 and 16 g/10 min at 260 °C for vPA12/CF and rPA12/CF, respectively. This behavior may be attributed, on the one hand, to the shortening of the CFs in the recycled composite as shown in Table S2 and, on the other hand, to the polymer chain scission that may result in a reduction in the average molecular weight [36,68].

In general, it has been reported that polymers with an MFI below 5 g/10 min can be difficult to extrude by 3D printing using the FFF technique [85,86,105,106,107,108]. In this case, the melted filament flows too slowly, strains the extruder motor, and causes filament grinding, eventually leading to under-extrusion. Above 20 g/10 min, the melted filament is too fluid [109,110], leading to poor control over part details and the occurrence of various defects during 3D printing, such as stringing (unsightly thin threads), over-extrusion, warping/curling/deformation, surface roughness, or bridging/sagging defects [1,111,112], as the material does not have time to crystallize quickly enough. Therefore, for FFF of thermoplastic materials, the recommended MFI values are between 5 and 20 g/10 min, providing a compromise between the flow of the melted filament through the nozzle and the ability to retain the deposed layer shape without nozzle clogging or any other printing defect.

From a processing perspective, a printing temperature of 250 °C will ensure a good flow behavior for both vPA12/CF and rPA12/CF during 3D printing. However, MFI tests are conducted at low shear rates and, thus, are related to the zero-shear viscosity rather than the flow in the nozzle. Thus, the selection of nozzle temperature must also be related to the flow at higher shear rates specific to the 3D printing (100–1000 s^−1^).

**Figure 7:**
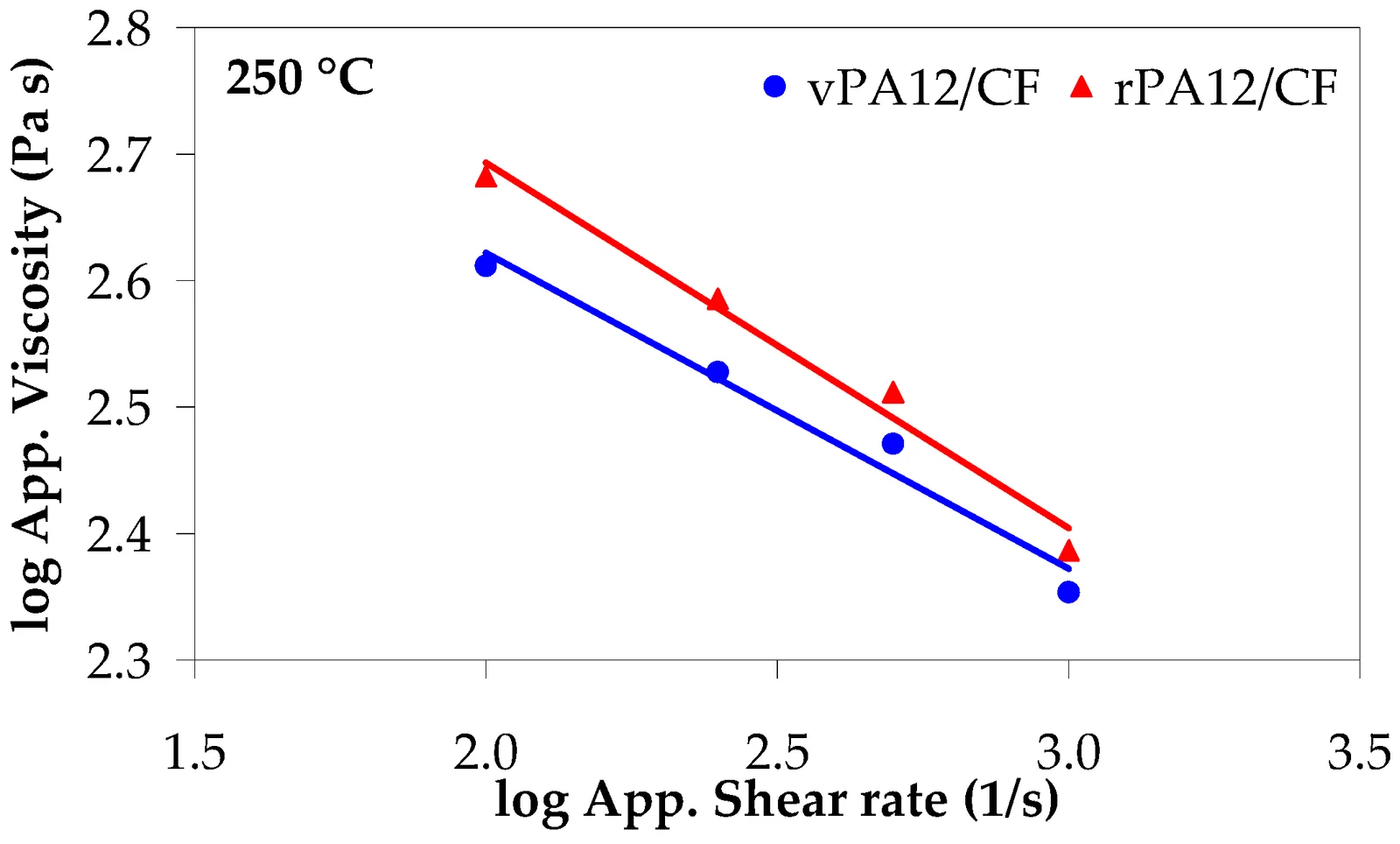
PA12/CF app. viscosity vs. app. shear rate.

To further investigate the flow behavior at 250 °C under high shear rates, Figure 7 presents the apparent melt shear viscosity curve, while the flow parameters based on the power-law model are presented in Table 5. A smaller flow index (*n*) indicates that the viscosity is more sensitive to changes in shear rates, while a lower flow consistency index (K) indicates better flowability. The rPA12/CF composite has a higher viscosity across the entire measured shear rate range compared to the vPA12/CF composite. However, both the virgin and recycled PA12/CF composites exhibit shear-thinning behavior, as indicated by the flow index *n* less than 1 [113]. For the recycled composite, the n value is at its minimum (0.71), while K is at its maximum (1866.81 Pa s^n^), indicating that the recycled composite displays an increased dependence on shear rate and lower flowability. This could be correlated with the CF length in the recycled composite, which is less concentrated and homogeneous (Table S2).

The contradictory observations in MFI and apparent melt shear viscosity can typically be explained by composite structural changes taking place simultaneously during mechanical recycling (shredding, compounding, re-extrusion) of PA12/CF composite, such as fiber fragmentation, chain scission, or solid-state post-condensation (chain extension) [114]. The increase in the MFI of the recycled composite might be related to fiber shortening and polymer chain scission. Shorter fibers have a lower aspect ratio and become less entangled at low shear rates, packing more easily into the melt [115]. Moreover, the PA12 matrix undergoes breakage of the long polymer chains into shorter segments [68,116]. This reduces flow resistance during MFI testing, allowing the rPA12/CF to flow faster and, thus, yielding a higher MFI value than the vPA12/CF (13 vs. 12 g/10 min). On the other hand, polyamides are condensation polymers [117] that can experience post-condensation (chain extension), branching, or cross-linking when exposed to multiple thermal cycles. At high shear rates, the newly cross-linked or branched polymer network becomes entangled around the CFs, increasing the overall melt shear viscosity across the 100 to 1000 s^−1^ spectrum.

### 3.5. Filament Extrusion and 3D-Printing Parts Quality

**Figure 8:**
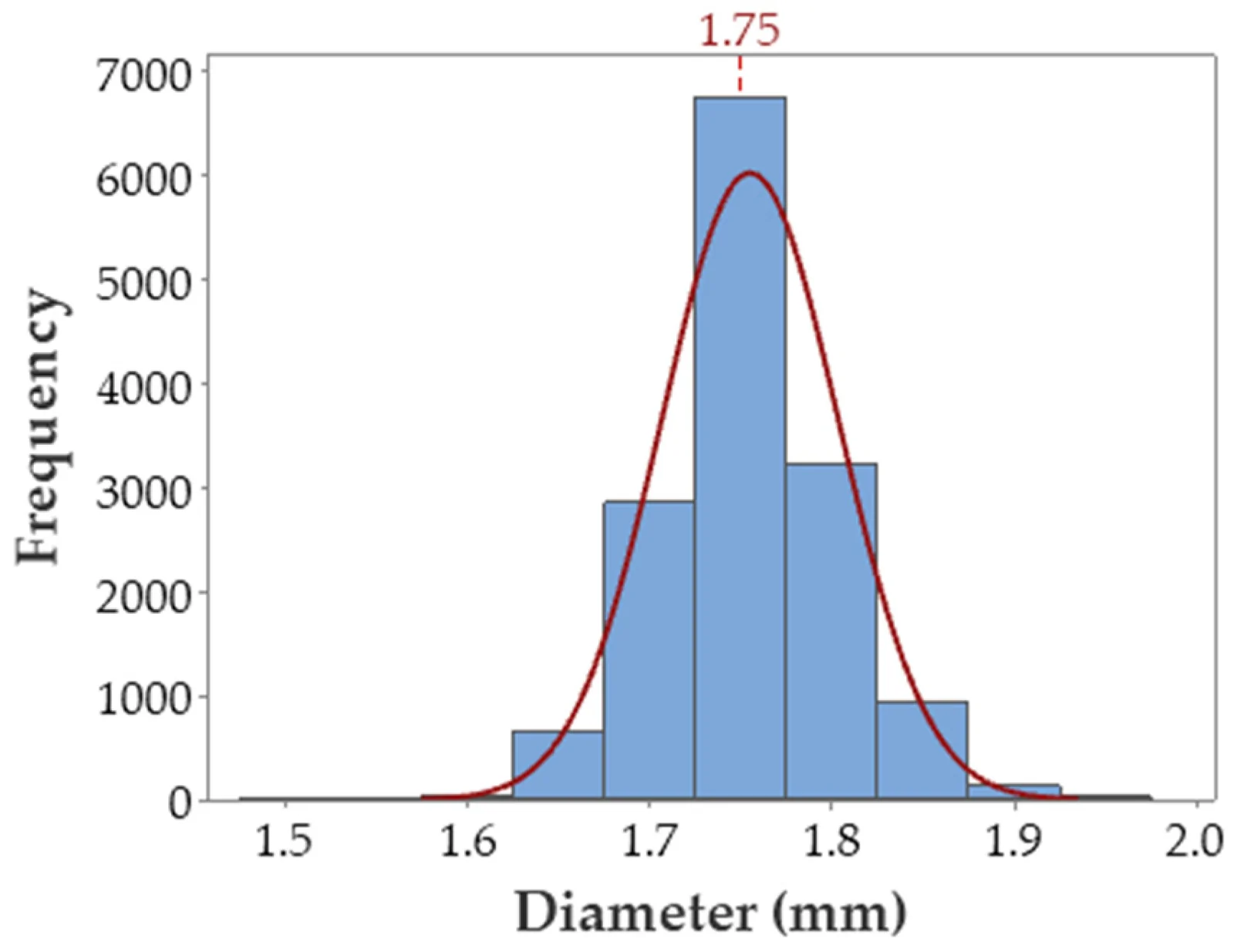
Diameter histogram of the rPA12/CF 3D extruded filament.

Figure 8 presents the diameter of the rPA12/CF filament measured during the filament extrusion process. It can be seen that the measured diameter has a normal distribution with an average value of 1.755 mm and an SD of 0.048 mm.

Table S1 from the Supplementary Materials presents the printability metrics of the 3D-printed PA12/CF composite samples. The analysis of the results shows that the 3D-printed parts made of vPA12/CF exhibited lower relative mass (weight) errors than those made of rPA12/CF. For example, the relative mass error of the vPA12/CF three-point bending sample 3D-printed with a 45° orientation was 0.9%, compared with 9.25% for the corresponding rPA12/CF sample. Overall, the vPA12/CF 3D-printed parts have an average mass relative error of 1.64%, whereas the corresponding value for rPA12/CF was 4.93%.

Regarding the UI in length and width, all types of 3D-printed samples made from virgin and recycled PA12/CF composite exhibited very good uniformity, with values close to 100 ± 2%. In contrast, the UI calculated for thickness/height (along the *Z*-axis) was consistently higher than 100% for the dog-bone and strip samples. For the dog-bone samples, the UI ranged from 102 to 107% for vPA12/CF and from 102 to 111% for rPA12/CF. For the strip samples, the corresponding values ranged from 108 to 115% and from 109 to 112%, respectively. On the other hand, the UI values calculated for the thickness/height of all three-point bending and compression samples were close to 100 ± 2%.

Overall, the UI results indicate that, although the 3D-printed dog-bone and strip samples exhibited noticeable deviations in thickness/height from their nominal dimensions, the remaining dimensions were reproduced with an accuracy of approximately ± 2% by the FFF process. The deviations along the *Z*-axis observed for the dog-bone and strip samples are likely associated with process-related factors (e.g., extrusion variability, layer-by-layer deposition, variations in layer height or printer calibration) and the relatively low thickness of these parts (1 and 2 mm), rather than with the mechanical recycling of the PA12/CF composite.

### 3.6. Microstructural Properties

**Figure 9:**
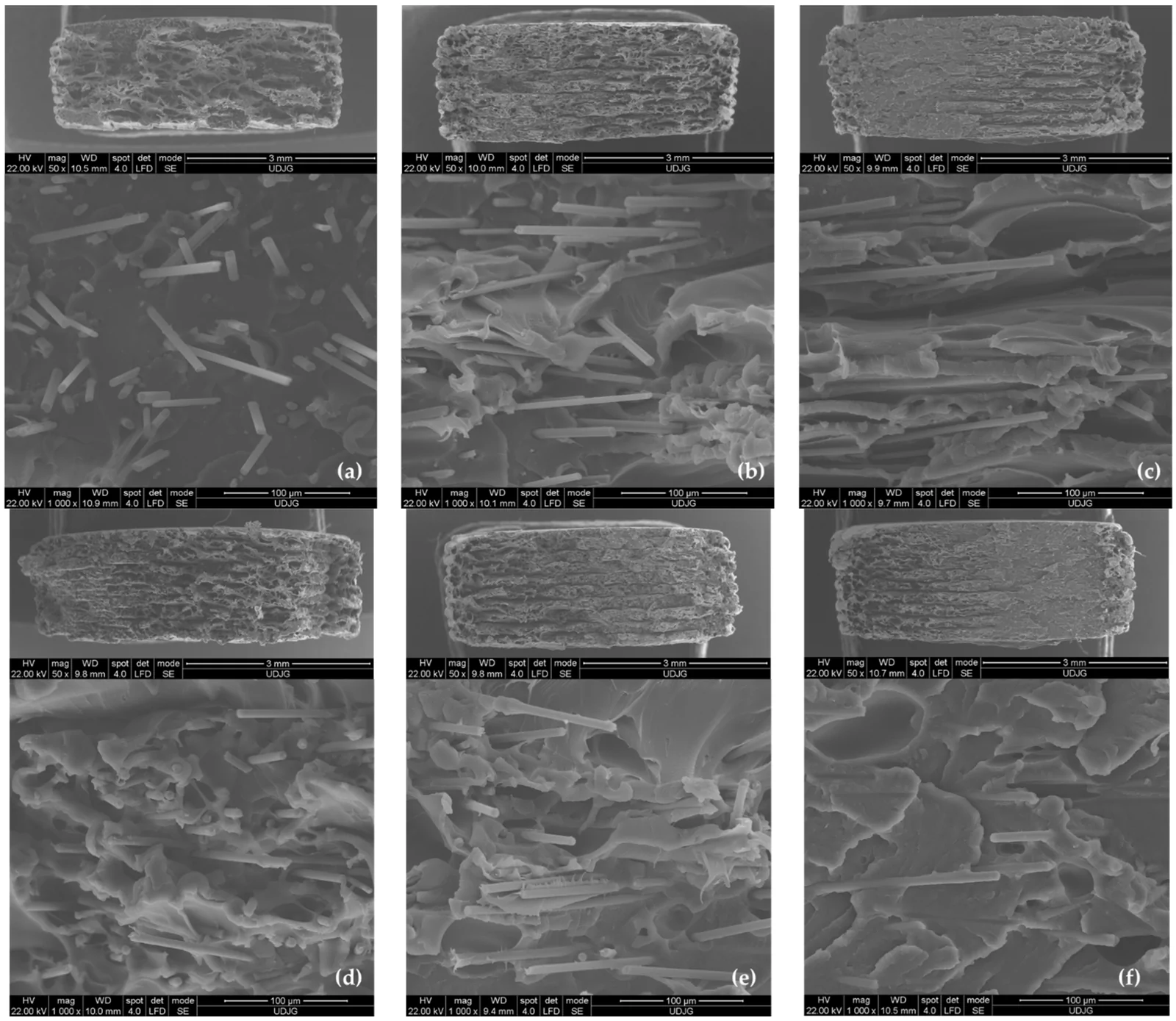
SEM images of the cross-section of the vPA12/CF 3D-printed with (**a**) 0°, (**b**) ±45°, and (**c**) 90° orientation; and rPA12/CF 3D-printed with (**d**) 0°, (**e**) ±45°, and (**f**) 90° orientation after tensile testing.

Figure 9 presents the SEM micrographs of the vPA12/CF and rPA12/CF composites on the tensile testing samples. The micrographs were obtained from the tensile samples printed with three orientations (0°, ±45°, and 90°), highlighting the influence of the printing strategy on the microstructural features and fracture mechanisms.

At low magnification (×50), in all printing orientations, the SEM images show a poros-like and layered structure that is typical for the 3D-printing process of composites filled with CFs. The layers and the resulting interfaces are clearly visible, indicating good bonding between adjacent layers. No significant voids, lack-of-fusion, or large discontinuities were observed within the examined regions.

At higher magnification (×1000), the SEM images clearly reveal the distribution and orientation of the CFs within the PA12 matrix as a function of printing orientation. The CFs appear as tubes with diameters ranging approximately from 5 to 10 μm. Moreover, micrographs indicate debonding between CFs and the PA12 matrix; however, there are very few CF pull-outs, indicating good adhesion between the matrix and the CFs, even for the recycled composite. For the samples 3D-printed with 0° orientation (Figure 9a,d), CFs are aligned perpendicular to the fractured surface (e.g., along the deposition orientation), as the CF ends are visible on the fractured surface. For the samples 3D-printed with ±45° orientation (Figure 9b,e), the CF orientation reflects the alternating layer pattern characteristic of the 3D printing strategy. For the samples with 90° orientation (Figure 9c,f), the CFs are predominantly aligned along the fracture surface, consistent with the transverse printing orientation with respect to the loading direction.

Regarding dispersion, the CFs appear relatively uniformly distributed throughout the PA12 matrix, with no evidence of significant agglomeration. A comparison of the virgin and recycled PA12/CF suggests that mechanical recycling and reprocessing by 3D printing did not significantly affect dispersion or distribution of CFs within the PA12 matrix.

A comparison between the vPA12/CF and rPA12/CF specimens reveals no major qualitative differences in CFs dispersion, CFs orientation, porosity, or fracture morphology. In both composites, the CFs remain uniformly distributed and maintain a preferential orientation dictated by the 3D printing process. The small number of CF pull-outs indicates good adhesion between the PA12 matrix and CFs.

### 3.7. Mechanical Properties

#### 3.7.1. Tensile Properties

**Figure 10:**
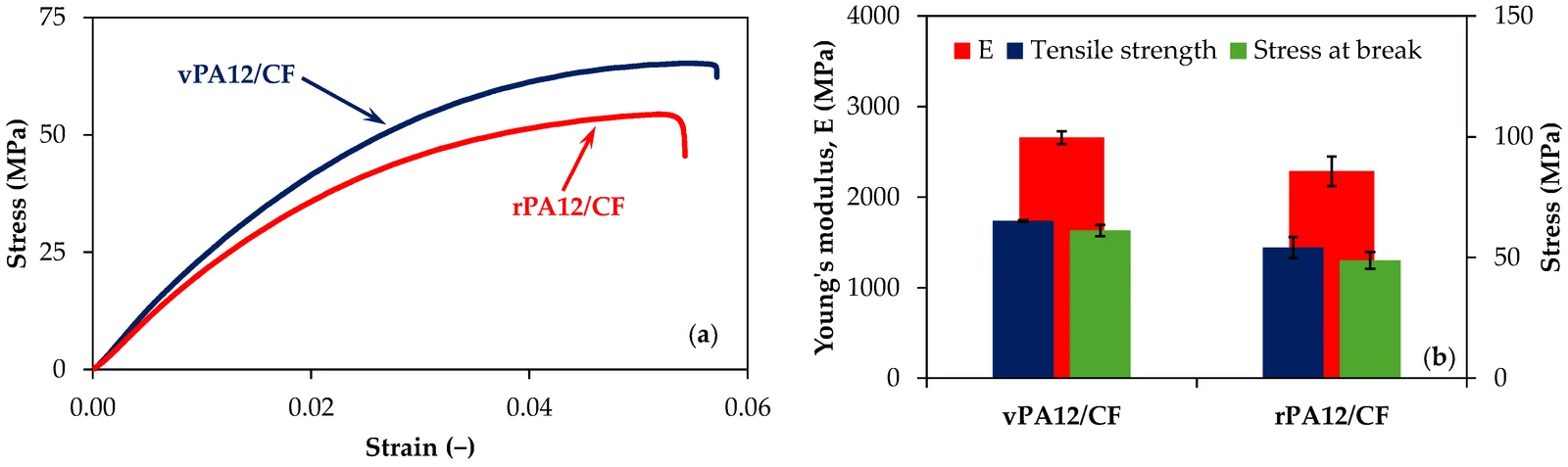
Tensile stress–strain curves (**a**) and tensile properties of the filament (**b**).

Figure 10a shows the tensile stress–strain curves for the vPA12/CF and rPA12/CF filaments, while the corresponding mechanical properties (e.g., Young’s modulus, tensile strength, and stress at break) are presented in Figure 10b. The tensile stress–strain curve indicates that the recycled filament has a lower load-bearing capacity as compared with the virgin filament.

Figure 10b indicates that the vPA12/CF filament has a Young’s modulus of about 2655 MPa, a maximum tensile stress of 65 MPa and a stress at break of 61 MPa. The rPA12/CF filament retains about 86% of the Young’s modulus (2285 MPa), 83% of the tensile stre88,89,40,90ngth (54 MPa), and 80% of the stress at break (49 MPa) of the vPA12/CF composite. Despite the decrease in mechanical properties, the rPA12/CF filament remains of interest for integration into the circular economy, as it retains a substantial proportion of the mechanical performance of vPA12/CF.

**Figure 11:**
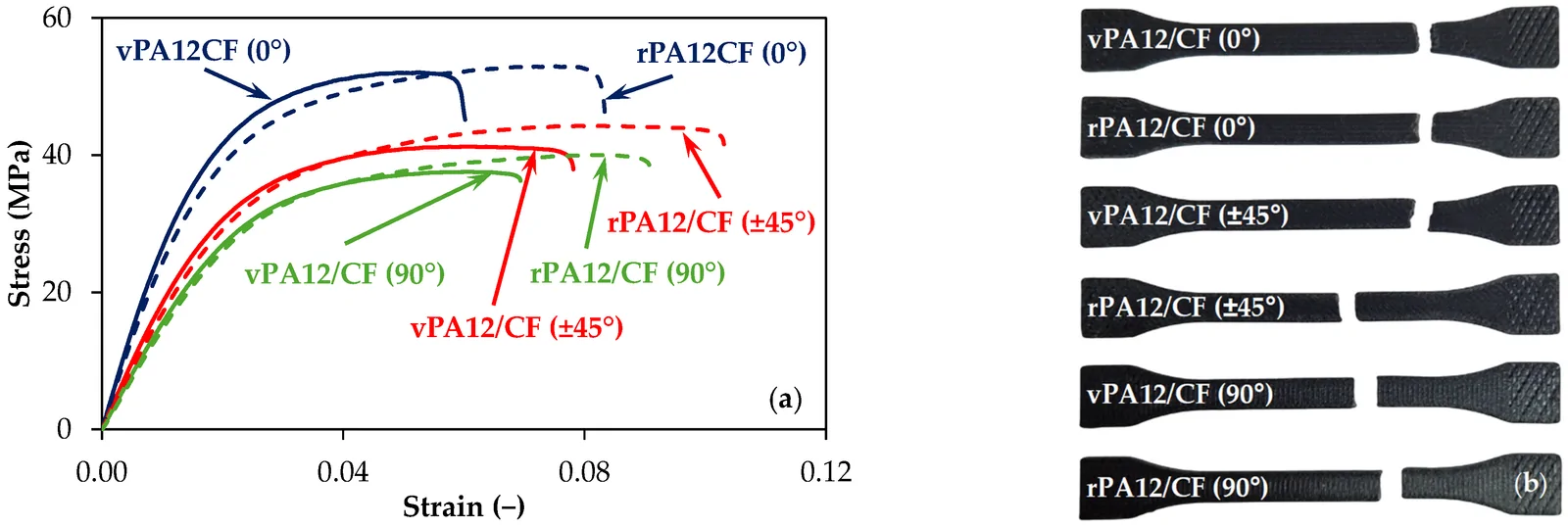
Tensile stress–strain curves of the 3D-printed PA12/CF dog-bone samples (**a**) and the corresponding tested samples (**b**).

**Figure 12:**
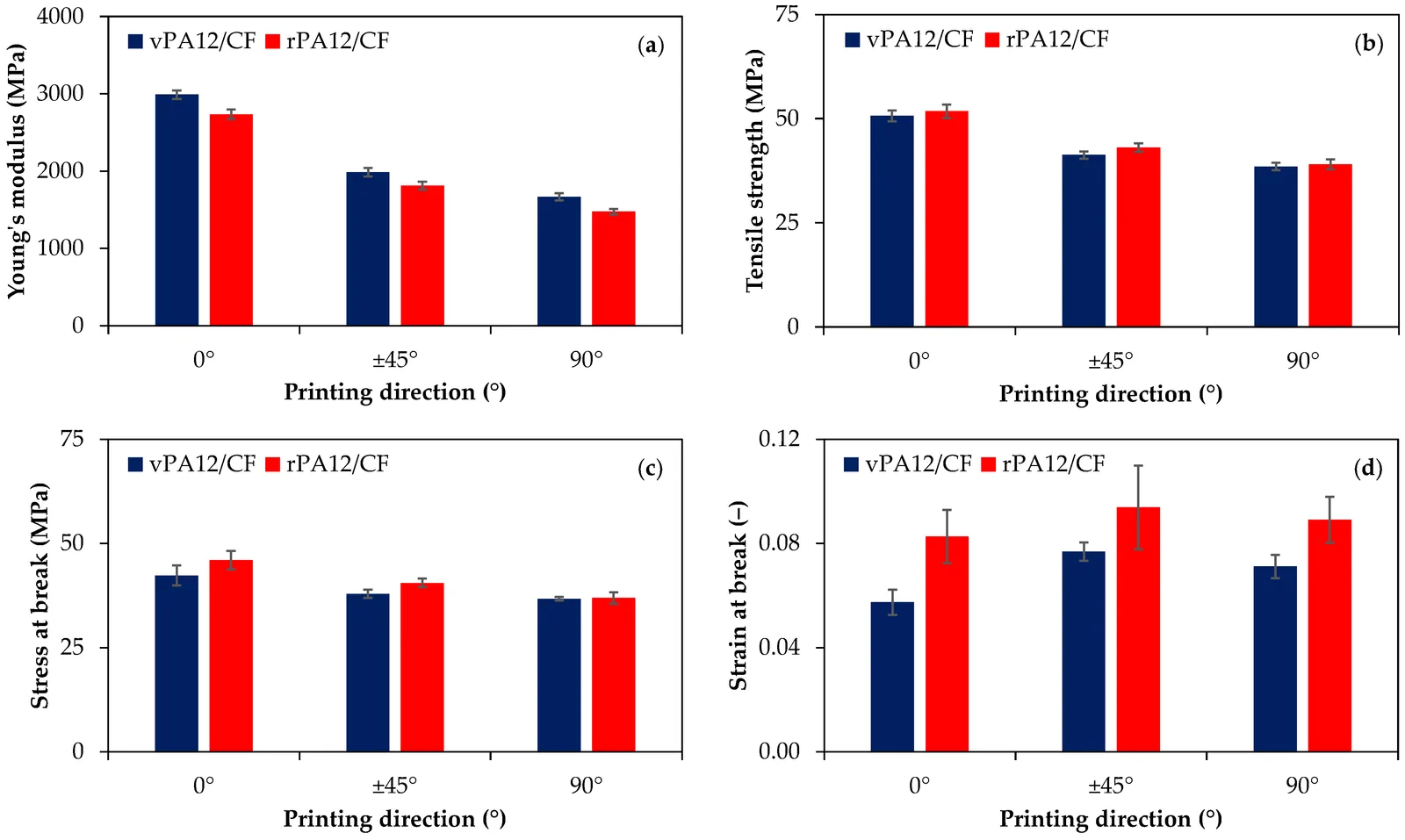
Tensile properties of 3D-printed the PA12/CF dog-bone samples: (**a**) Young’s modulus, (**b**) tensile strength, (**c**) stress at break, and (**d**) strain at break.

The tensile stress–strain curves of the 3D-printed PA12/CF dog-bone samples are presented in Figure 11a, while the fractured samples are presented in Figure 11b. The corresponding mechanical properties (e.g., Young’s modulus, tensile strength, stress and strain at break) are summarized in Figure 12.

The tensile stress–strain curves show a clear influence of the printing orientation on the mechanical behavior of PA12/CF composites. As shown in Figure 11a, because of the alignment of the CFs along the loading direction, the samples printed with 0° exhibit the highest mechanical properties (Young’s modulus, tensile strength, and stress at break of 2987 MPa, 51 MPa, and 42 MPa, respectively, for vPA12/CF and 2733 MPa, 52 MPa, and 46 MPa, respectively, for rPA12/CF).

The printing orientation controls not only the spatial distribution of CFs but also the fracture mechanisms of the 3D-printed PA12/CF composite. Samples printed with 0° orientation have the highest tensile strength and stiffness, while the samples printed with 90° and ±45° show reduced strength due to weaker interlayer bonding and diagonal stress distribution. These results are supported by the SEM images, indicating that in the samples 3D-printed with 0° orientation, the CFs are predominantly oriented along the deposition direction, which is parallel to the tensile loading direction, enhancing the stress transfer between the fibers and matrix. For the samples printed with 90° orientation, the CFs are predominantly oriented perpendicular to the loading direction, and the stress concentrates on the interlayer bonds between layers rather than the polymer matrix. In the case of samples printed with ±45° orientation, the tensile strength falls between those of the 0° and 90°, as interlayer sliding and matrix shear may occur.

The vPA12/CF Young’s modulus, tensile strength, and stress at break of samples printed with a 90° orientation decreased by about 44, 24, and 13%, respectively, compared to samples printed with a 0° orientation. The 3D-printed layers aligned parallel to the loading direction (0°) transfer the applied load more efficiently than those oriented perpendicular to the loading direction (90°). The mechanical response is governed primarily by the weaker interlayer bonding [2].

Regardless of orientation, mechanical recycling resulted in a moderate reduction in the Young’s modulus of the rPA12/CF composite. The Young’s modulus of samples printed with 0°, ±45°, and 90° orientations decreased by approximately 9, 9, and 12%, respectively, compared with the corresponding vPA12/CF samples. This reduction suggests a slight decrease in composite stiffness, which may be associated with polymer chain scission during processing and partial shortening or damage of the CFs during grinding and extrusion [36,68]. In contrast, depending on orientation, the tensile strength, stress at break, and strain at break improved after recycling, with increases of up to 4, 9, and 44%, respectively, compared to the 3D-printed vPA12/CF composite. The increase in strain at break indicates that the rPA12/CF exhibited better ductile behavior before fracturing as compared to the vPA12/CF composite.

Statistical analysis (Table S3 of Supplementary Materials) shows that the recycling process had a statistically significant effect (*p*-Value < 0.05) on Young’s modulus and strain at break, with contributions of 3 and 70%, respectively. In contrast, orientation had been found to have a very significant effect (*p*-Value < 0.01) on Young’s modulus and tensile strength, with its contribution to the overall variation being above 96%. This indicates that orientation is the dominant factor governing the tensile behavior of the 3D-printed PA12/CF composite.

**Figure 13:**
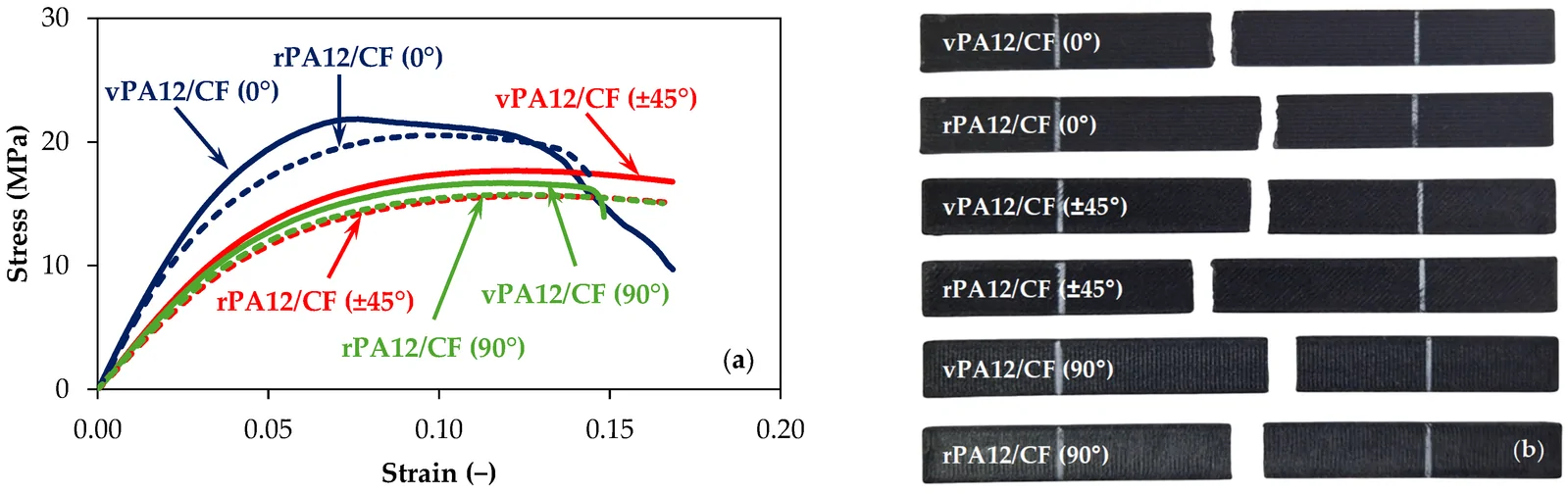
Tensile stress–strain curves of the 3D-printed PA12/CF strip samples (**a**) and the corresponding tested samples (**b**).

**Figure 14:**
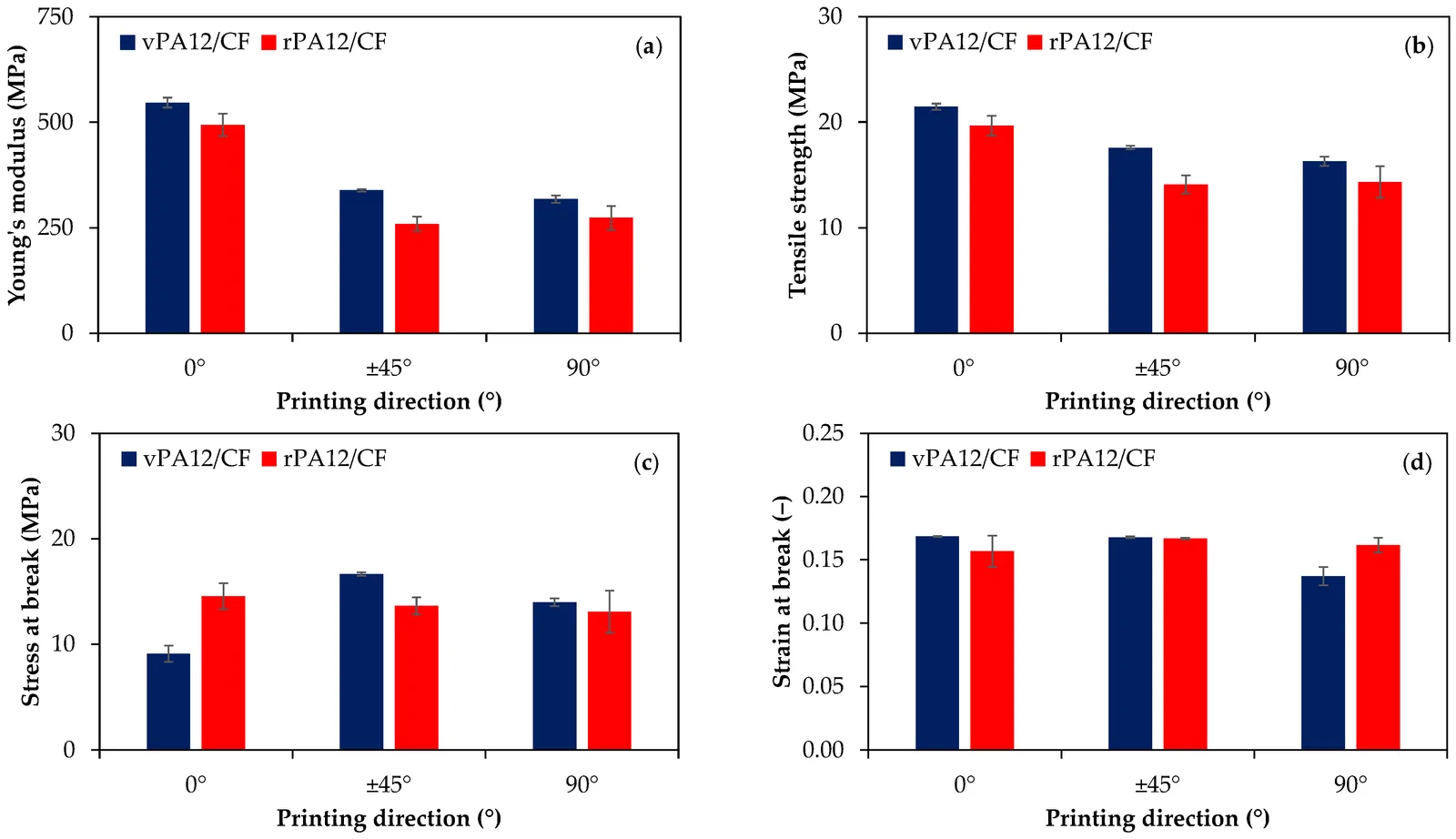
Tensile properties of the 3D-printed PA12/CF strips samples: (**a**) Young’s modulus, (**b**) tensile strength, (**c**) stress at break, and (**d**) strain at break.

Figure 13 shows the tensile stress–strain curves of the 3D-printed PA12/CF strip samples, while the corresponding mechanical properties are presented in Figure 14.

Similar to the dog-bone samples, the 3D-printed vPA12/CF strips with 0° orientation exhibited the highest Young’s modulus and tensile strength, up to 550 MPa and 21 MPa, respectively, while the corresponding 90° orientation was up to 320 MPa and 16 MPa. On the other hand, the 3D-printed rPA12/CF strips have lower Young’s modulus and tensile strength regardless of orientation. For example, the Young’s modulus of the rPA12/CF strips printed with 0°, ±45°, and 90° orientations decreased by 10, 24, and 14%, respectively, while the tensile strength decreased by 8, 20, and 12%, respectively, compared to vPA12/CF. The stress at break of the rPA12/CF strips shows an orientation-dependent behavior compared to vPA12/CF. At 0°, the stress at break increases by approximately 60%, from 9.0 to 14.5 MPa, whereas it decreases by approximately 18 and 6% for the ±45° and 90° orientations, respectively. The strain at break exhibits a different trend, decreasing slightly by approximately 7 and 1% for the 0° and ±45° orientations, respectively, while increasing by approximately 18% for the 90° orientation.

Statistical analysis, as shown in Table S4 of the Supplementary Materials, indicates that orientation is the most significant factor (*p*-Value < 0.05) affecting the Young’s modulus and tensile strength of the 3D-printed strips, with contributions of 92 and 78%, respectively. Mechanical recycling is also confirmed as a significant factor affecting the Young’s modulus and tensile strength, with contributions of 7 and 21%, respectively. Therefore, printing orientation remained the main factor controlling the tensile performance of the 3D-printed composites, whereas mechanical recycling had a secondary but significant effect.

#### 3.7.2. Three-Point Bending

**Figure 15:**
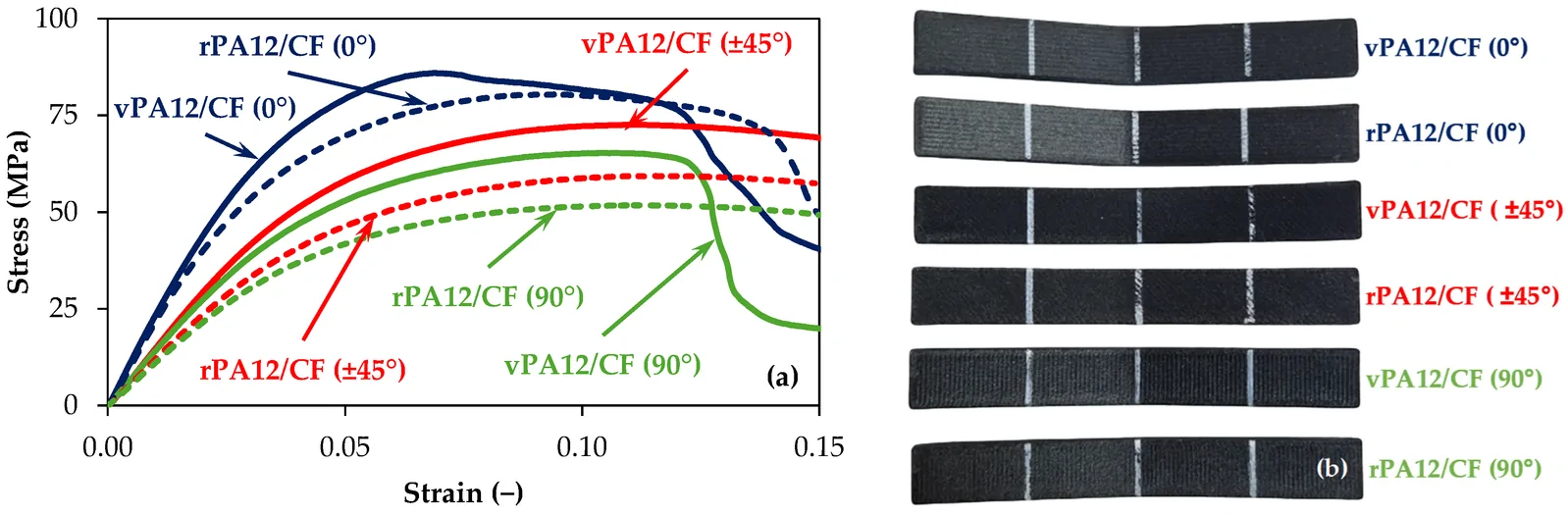
Bending stress–strain curves of the 3D-printed PA12/CF samples (**a**) and tested samples (**b**).

Representative bending stress–strain curves of the 3D-printed PA12/CF samples are presented in Figure 15a, while the tested samples after bending are shown in Figure 15b. It should be noted that all samples were loaded up to a deflection of 10 mm, corresponding to a strain of approximately 15%, and none of the samples exhibited fracture during testing. As expected, the samples printed with 0° orientation exhibited the highest bending performance. For example, the vPA12/CF composites show a bending modulus of 2400 MPa and a bending strength of 85 MPa, while for the 90° printing orientation, these mechanical properties decreased by approximately 41 and 23%, respectively.

**Figure 16:**
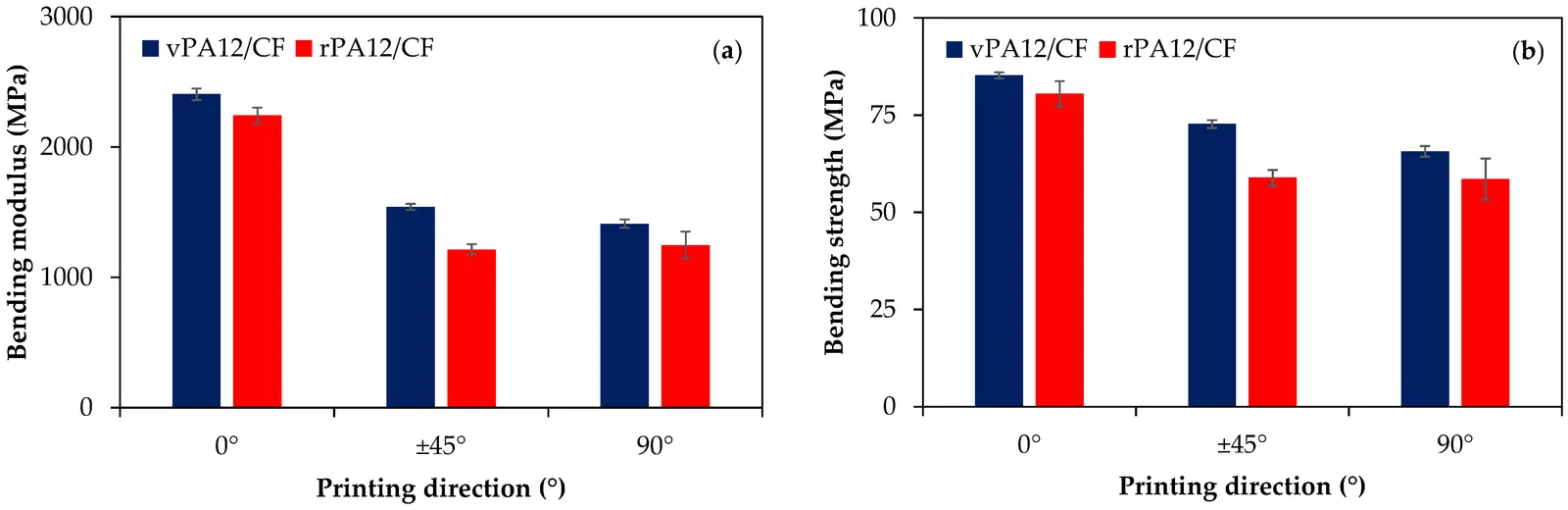
Bending properties of the: bending modulus (**a**) and bending strength (**b**) of 3D-printed PA12/CF samples.

As can be observed in Figure 16, mechanical recycling led to a small reduction in both bending modulus and bending strength, regardless of the printing orientation. Compared to the vPA12/CF composite, the bending modulus of the rPA12/CF decreased by 7, 21, and 12% for the 0°, ±45°, and 90° printing orientations, respectively, while the bending strength decreased by 6, 19, and 11%, respectively. It can be seen that the ±45° orientation suffers the most significant degradation in both stiffness (21%) and strength (19%), while the 0° orientation is the most stable.

Statistical analysis (Table S5 of the Supplementary Materials) confirmed that the orientation had a statistically significant effect on the bending properties (*p*-Value < 0.05), accounting for approximately 94% of the overall variation. Recycling had no statistically significant effect on either the bending modulus or the bending strength (*p*-Value > 0.05).

#### 3.7.3. Compression

**Figure 17:**
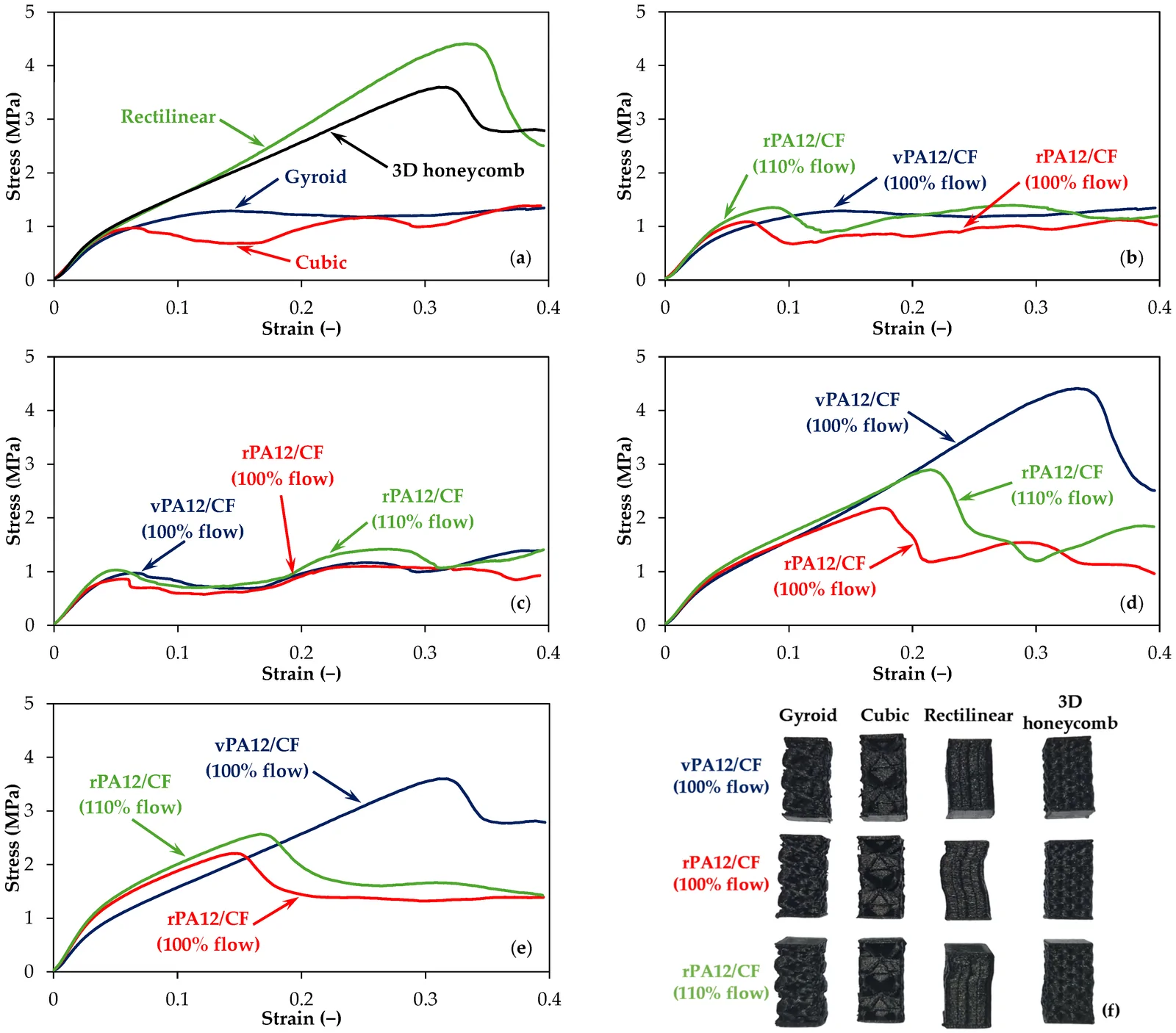
Compression stress–strain curves of 3D-printed PA12/CF samples (**a**), gyroid pattern (**b**), cubic pattern (**c**), rectilinear pattern (**d**), 3D honeycomb pattern (**e**) and tested samples (**f**).

**Figure 18:**
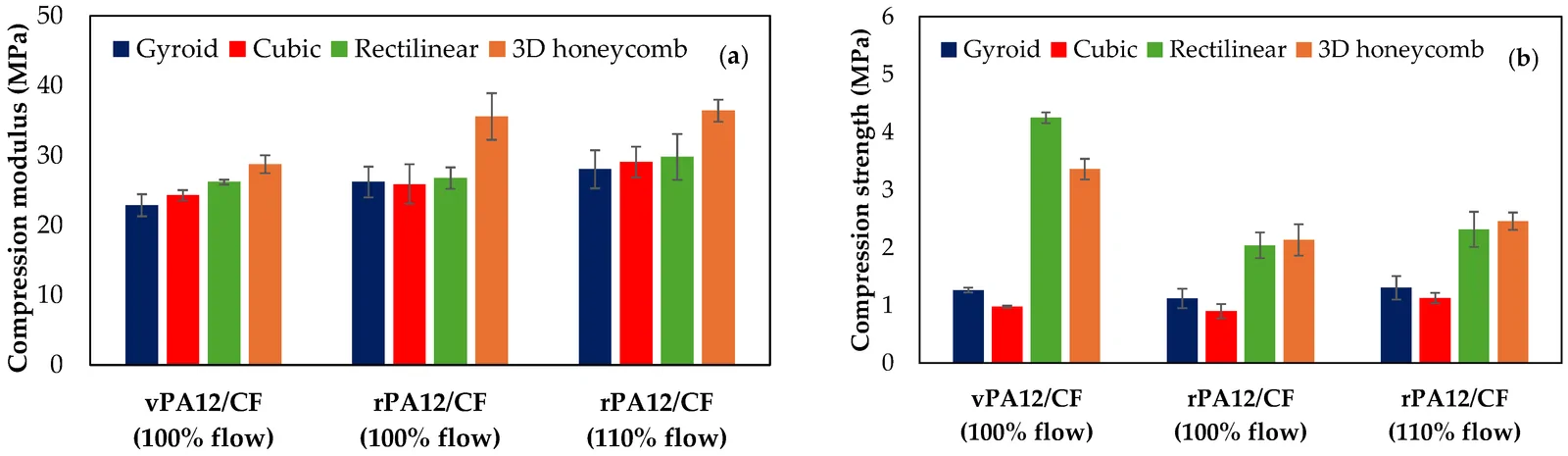
Compression properties of the 3D-printed PA12/CF samples: Young’s modulus (**a**), and compression strength (**b**).

Figure 17a–e compare the compression stress–strain behavior of the PA12/CF samples as a function of pattern type, infill pattern, flow rate, and recycling, while the corresponding compressive modulus and compressive strength are summarized in Figure 18. All samples (Figure 17f) were compressed up to 10 mm, corresponding to a strain of approximately 40%. As shown in Figure 17a, among the vPA12/CF samples 3D-printed with a 100% flow rate, the rectilinear pattern exhibited the highest compressive strength, followed by the 3D honeycomb pattern, while the cubic and gyroid patterns exhibited the lowest compressive strength.

Since all samples were printed with 15% infill density, the difference between the compressive modulus (Figure 18a) and compressive strength (Figure 18b) of the PA12/CF composite can be attributed to the distinct deformation and failure mechanisms associated with each 3D-printed pattern. While the compressive modulus is primarily associated with the initial load-transfer efficiency and resistance to deformation, the compressive strength depends on how the cellular pattern responds to increasing deformation, including local stress concentration, cell-wall bending and buckling, load redistribution, and the resulting failure mode [118,119,120].

The 3D-printed structures with a honeycomb pattern are the most rigid geometric structures because when the hexagonal structure is extruded, the intersecting angles create the highest geometric rigidity (Figure 18a). On the other hand, the 3D-printed structures with a rectilinear pattern exhibit the highest compressive strength (Figure 18b) because the rectilinear pattern consists of long, continuous vertical walls that resist crushing when the load is aligned with the internal 3D-printed lines.

Regardless of the infill pattern, the mechanical recycling of the PA12/CF composite caused an increase in the compression modulus compared to its virgin counterparts (Figure 18a). The rPA12/CF samples with a 3D honeycomb pattern exhibit the largest increase of approximately 24% (36 MPa), followed by the gyroid pattern at 15% (26 MPa), and the cubic and rectilinear patterns of 7% (26 MPa) and 2% (27 MPa), respectively, compared to their virgin counterparts. Instead, from Figure 18b, it can be observed that 3D printed rPA12/CF composites exhibit lower compressive strength, particularly in the case of the rectilinear and 3D honeycomb patterns, where the rPA12/CF samples lost about 52% (2.04 MPa) and 37% (2.13 MPa) in compression strength compared to their vPA12/CF counterparts.

During the 3D printing process, compression properties can be controlled by increasing the extrusion flow rate. Figure 17 and Figure 18 show that increasing the flow rate from 100 to 110% partially compensated for the reduction in the rPA12/CF compressive strength. The rPA12/CF samples with gyroid and cubic patterns printed with a 110% flow rate have better compressive strength 1.30 MPa and 1.13 MPa, respectively, compared to vPA12/CF counterparts printed with 100% flow rate. Although the rPA12/CF rectilinear and 3D honeycomb patterns printed with 110% did not fully recover to the values obtained for the virgin composite, the compressive strength shows an encouraging improvement with increasing the flow rate to higher values of 110%.

The statistical analysis (Table S6 of the Supplementary Materials) indicated that the infill pattern is the most statistically significant factor (*p*-Value < 0.05) affecting both the compressive modulus and compressive strength, with a contribution of approximately 63% and 69% to the overall results, respectively. The recycling process significantly affected only the compressive modulus, with a contribution of up to 26%. Even though the flow rate was increased from 100 to 110% to compensate for the reduction in the compressive properties, from a statistical point of view, this increase of 10% in flow rate does not have a statistically significant effect (*p*-Value > 0.05).

## 4. Conclusions

This paper presents a comprehensive investigation into the mechanical recycling of small-volume PA12/CF waste using FFF. vPA12/CF composite waste was mechanically recycled and reprocessed by filament extrusion and FFF. The effect of mechanical recycling and subsequent FFF reprocessing was assessed in detail by changes in the physical, chemical, microstructural, rheological, carbon fiber length, and mechanical properties. Based on the experimental results, the following conclusions have been drawn:

• Mechanical recycling did not affect the solid density of the rPA12/CF composite, but it led to a decrease in the mean fiber length (from 0.203 mm in vPA12/CF to 0.147 mm in rPA12/CF). Moreover, about 25% of the CF lengths of the rPA12/CF have been crushed into micro-particles under 0.080 mm, compared to 0.120 mm in the vPA12/CF filament.
• FT-IR analysis confirmed that PA12/CF exhibits very good chemical stability with recycling, showing only one new functional group with a low-intensity peak around 1738 cm^−1^.
• DSC analysis shows good thermal stability of the rPA12/CF composites, with minor changes (1–2 °C) in the glass transition, crystallization, and melting temperatures. The 15% increase in the crystallinity degree of the recycled filament correlates with the change in the CF length that promotes the nucleation process.
• While the MFI suggested that the rPA12/CF composite exhibited improved flowability with the optimal nozzle temperature of 250 °C, the capillary rheological testing indicated that the rPA12/CF composite has a higher viscosity compared to the vPA12/CF composite across the entire measured shear rate range (100–1000 s^−1^); however, the rPA12/CF composite exhibits better shear-thinning behavior.
• The diameter of the rPA12/CF filament is consistent with the standard specification (1.755 mm and an SD of 0.048), while retaining over 80% of the mechanical properties of the vPA12/CF filament.
• SEM micrographs revealed good interlayer bonding, uniform CF distribution, and no significant defects in either virgin or recycled PA12/CF composites.
• The mechanical recycling process caused reductions in the tensile stiffness of rPA12/CF; with the 0° printing orientation retaining at least 90% of the Young’s modulus or bending modulus values of the vPA12/CF counterparts. Tensile strength, stress at break, and strain at break of the rPA12/CF tensile samples increased by about 10% after the recycling process, as shorter rCFs improve the ductility and crystallinity. Bending strength, however, was negatively affected by recycling, showing a decrease of up to 20%, depending on the printing orientation.
• The compressive response was strongly dependent on the infill pattern, with the rectilinear and 3D honeycomb configurations providing the highest compressive strength and modulus, respectively.

In conclusion, the experimental results demonstrate the potential of FFF 3D printing for reprocessing mechanically recycled PA12/CF composite waste, particularly for small amounts of composite waste. The current framework will be further extended to include mechanical recycling, including multi-cycle loop recycling, and to examine other properties such as molecular weight and mechanisms of degradation at the polymer chain level. In addition, the recycled PA12/CF filament will be further used for 3D printing of more complex industrial parts where the balance between the cost and properties of the end-used part justifies the use of recycled composite.

## Figures and Tables

**Table 1:** 3D-printing conditions.

	Sample Type	Dog-Bone	Strip	Three-Point Bending	Compression
Parameter					
Nozzle diameter (mm)		0.4	0.4	0.4	0.4
Nozzle temperature (°C)		250	250	250	250
Bed temperature (°C)		90	90	90	90
Layer height (mm)		0.2	0.2	0.2	0.2
Flow ratio (%)		95	95	95	95
Orientation (°)		0; ±45; 90	0; ±45; 90	0; ±45; 90	0
Perimeters (-)		1	1	1	0
Infill density (%)		100	100	100	15
Infill pattern		Rectilinear	Rectilinear	Rectilinear	3D honeycomb; Cubic; Gyroid; Rectilinear
Printing speed (mm)		20	20	20	20
Fan speed (%)		20	20	20	20
Top solid layers (-)		-	-	-	4
Bottom solid layers (-)		-	-	-	4
Seam position		Aligned	Aligned	Aligned	-
Brim width (mm)		-	-	-	10

**Table 2:** Solid density of vPA12/CF and rPA12/CF.

Samples From	Density (g/cm^3^)
Filament (F)	1.0094 ± 0.0067
Recycled filament (rF)	1.0097 ± 0.0186
Printed samples (PS)	0.9445 ± 0.0104
PS from rF (rPS)	0.9368 ± 0.0079

**Table 3:** Ash content of virgin and recycled PA12/CF composite.

PA12/CF (500 °C)	vPA12/CF		rPA12/CF	
	Weight (g)	Ash Content (%)	Weight (g)	Ash Content (%)
Initial sample	2.0402 ± 0.0184	-	2.0129 ± 0.0047	-
1st flame (30 min)	0.1314 ± 0.0021	6.44 ± 0.0806	0.1180 ± 0.0016	5.86 ± 0.0786
2nd flame (5 min)	0.1310 ± 0.0025	6.42 ± 0.0908	0.1172 ± 0.0016	5.82 ± 0.0773
3rd flame (5 min)	0.1297 ± 0.0026	6.36 ± 0.1044	0.1168 ± 0.0016	5.80 ± 0.0787
4th flame (5 min)	0.1289 ± 0.0029	6.32 ± 0.1110	0.1161 ± 0.0015	5.77 ± 0.0799

**Table 4:** Thermal properties of the PA12/CF composite.

PA12/CF		F	rF	PS	rPS
First Cooling	T_c_ (°C)	154.58 ± 0.59	156.87 ± 0.46	154.43 ± 0.29	156.30 ± 0.10
	T_g_ (°C)	52.02 ± 1.09	53.58 ± 1.32	53.24 ± 1.27	53.32 ± 0.35
Second Heating	T_m_ (°C)	176.53 ± 0.36	176.36 ± 0.36	176.52 ± 0.86	175.78 ± 0.01
	T_shoulder_ (°C)	167.81 ± 3.67	171.20 ± 0.70	170.17 ± 1.53	170.30 ± 0.12
	ΔH_m_ (J/g)	65.20 ± 3.17	74.85 ± 2.11	73.60 ± 3.21	71.11 ± 3.90
	X_c_ (%)	24.92 ± 1.21	28.61 ± 0.81	28.13 ± 1.23	27.18 ± 1.49

F—filament; rF—recycled filament; PS—printed samples; rPS—PS from rF.

**Table 5:** Solid density of vPA12/CF and rPA12/CF.

	Flow Consistency Index, K (Pa s^n^)	Flow Behavior Index, n (-)
vPA12/CF	1322.51	0.75
rPA12/CF	1866.81	0.71

## Data Availability

The original contributions presented in this study are included in the article/Supplementary Materials. Further inquiries can be directed to the corresponding author.

## Supplementary Materials

The following supporting information can be downloaded at: https://www.mdpi.com/article/10.3390/polym18202522/s1; Table S1. Printability of the PA12/CF composites samples, Table S2. Carbon fiber length in vPA12/CF and rPA12/CF composite filament, Table S3. Summary of the ANOVA of the tensile tests (dog-bone samples), Table S4. Summary of the ANOVA of the tensile tests (strip samples), Table S5. Summary of the ANOVA of the three-point bending tests, Table S6. Summary of the ANOVA of the compression tests.
