# Supplementary material for: 3D Printing of Mechanical Recycled PA12/CF Composite
Source: Polymers (Basel). 2026 Oct 15;18(20):2522. doi: 10.3390/polym18202522 (PMC13708906; doi:10.3390/polym18202522)
Supplement: Supplementary file 1 [file polymers-18-02522-s001.zip › polymers-4570845-supplementary.pdf]

polymers

MDPI

Supplementary Materials

3D Printing of Mechanical Recycled PA12/CF Composite

Ionut-Laurentiu Sandu [1,*] and Felicia Stan [1,*], Catalin Fetecau [1] and Andreea Botezatu [2]

[1] Center of Excellence Polymer Processing, "Dunarea de Jos" University of Galati, 47 Domneasca, 800 008 Galati, Romania; felicia.stan@ugal.ro (F.S.), catalin.fetecau@ugal.ro (C.F.)
[2] Department of Chemistry, Physics and Environment, Faculty of Sciences and Environment, "Dunarea de Jos" University of Galati, 111 Domneasca, 800 201 Galati, Romania; andreea.botezatu@ugal.ro (A.B.)
* Correspondence: laurentiu.sandu@ugal.ro (I.-L.S.), felicia.stan@ugal.ro (F.S.)

1. Tables

Table S1. Printability of the PA12/CF composites samples

Composite	3D-printed sample type	Orientation (°) / Infill pattern	Weight (g)	Relative error (%)	Length (mm)	Uniformity index (%)	Width (mm)	Uniformity index (%)	Thickness / Height (mm)	Uniformity index (%)
vPA12/CF	Tensile dog-bone	0	0.9134 ± 0.0041	4.29	75.23 ± 0.09	100.31	5.09 ± 0.01	101.88	2.03 ± 0.02	101.50
		±45	0.9371 ± 0.0027	1.81	74.93 ± 0.04	99.90	5.12 ± 0.02	102.40	2.05 ± 0.02	102.69
		90	0.9401 ± 0.0048	1.49	74.46 ± 0.08	99.28	5.14 ± 0.01	102.83	2.13 ± 0.03	106.56
	Tensile strip	0	0.9962 ± 0.0044	4.12	110.31 ± 0.04	100.28	10.04 ± 0.04	100.41	1.08 ± 0.02	108.38
		±45	1.0220 ± 0.0061	1.63	109.87 ± 0.04	99.88	10.05 ± 0.02	100.48	1.15 ± 0.02	115.13
		90	1.0296 ± 0.0059	0.90	109.09 ± 0.06	99.17	10.08 ± 0.02	100.76	1.11 ± 0.02	111.25
	Three-point bending	0	2.9170 ± 0.0124	3.49	80.35 ± 0.03	100.43	10.12 ± 0.04	101.16	4.03 ± 0.01	100.78
		±45	2.9951 ± 0.0142	0.90	79.89 ± 0.08	99.86	10.12 ± 0.01	101.19	4.05 ± 0.02	101.25
		90	3.0366 ± 0.0137	0.47	79.44 ± 0.08	99.29	10.11 ± 0.01	101.09	4.06 ± 0.04	101.56
	Compression (100% flow)	3D honeycomb	1.0355 ± 0.0091	0.43	12.91 ± 0.04	101.65	12.79 ± 0.05	100.71	25.29 ± 0.05	99.57
		Cubic	0.9231 ± 0.0074	0.74	12.81 ± 0.05	100.87	12.75 ± 0.03	100.39	25.27 ± 0.04	99.49
		Gyroid	0.9337 ± 0.0069	0.67	12.83 ± 0.04	101.02	12.78 ± 0.04	100.63	25.29 ± 0.04	99.57
		Rectilinear	0.9561 ± 0.0061	0.41	12.84 ± 0.03	101.10	12.74 ± 0.05	100.31	25.26 ± 0.06	99.45
rPA12/CF	Tensile dog-bone	0	0.9136 ± 0.0319	3.49	75.06 ± 0.06	100.08	5.07 ± 0.06	101.42	2.04 ± 0.03	101.75
		±45	0.9460 ± 0.0221	0.07	74.83 ± 0.04	99.77	5.08 ± 0.04	101.54	2.08 ± 0.06	104.20
		90	0.9585 ± 0.0190	1.26	74.36 ± 0.09	99.14	5.12 ± 0.05	102.30	2.21 ± 0.06	110.70
	Tensile strip	0	1.0114 ± 0.0255	1.85	110.14 ± 0.06	100.12	9.98 ± 0.05	99.80	1.09 ± 0.03	108.86
		±45	1.0526 ± 0.0098	2.15	109.75 ± 0.05	99.78	9.99 ± 0.03	99.87	1.12 ± 0.03	111.71
		90	1.0640 ± 0.0233	3.26	109.06 ± 0.15	99.15	10.03 ± 0.02	100.28	1.11 ± 0.02	111.00
	Three-point bending	0	2.7204 ± 0.1096	9.25	80.07 ± 0.07	100.09	9.84 ± 0.28	98.37	4.02 ± 0.02	100.46
		±45	2.7228 ± 0.0700	9.17	79.75 ± 0.03	99.68	9.95 ± 0.01	99.54	4.00 ± 0.01	100.11
		90	2.8020 ± 0.1519	6.53	79.16 ± 0.08	98.95	10.01 ± 0.03	100.10	4.03 ± 0.02	100.71
	Compression (100% flow)	3D honeycomb	0.9655 ± 0.0571	7.16	12.79 ± 0.06	100.71	12.74 ± 0.05	100.31	25.23 ± 0.02	99.33
		Cubic	0.8675 ± 0.0470	6.72	12.71 ± 0.12	100.08	12.71 ± 0.06	100.08	25.24 ± 0.02	99.37
		Gyroid	0.8662 ± 0.0384	6.86	12.75 ± 0.05	100.39	12.73 ± 0.05	100.24	25.24 ± 0.03	99.37
		Rectilinear	0.8903 ± 0.0329	6.28	12.82 ± 0.07	100.94	12.70 ± 0.04	100.00	25.32 ± 0.06	99.69
	Compression (110% flow)	3D honeycomb	1.0191 ± 0.0247	2.01	12.86 ± 0.05	101.26	12.78 ± 0.06	100.63	25.33 ± 0.03	99.72
		Cubic	0.9681 ± 0.0357	4.10	12.88 ± 0.08	101.42	12.80 ± 0.05	100.79	25.41 ± 0.05	100.04
		Gyroid	0.9068 ± 0.0656	2.49	12.80 ± 0.10	100.79	12.74 ± 0.08	100.31	25.37 ± 0.05	99.88
		Rectilinear	0.9590 ± 0.0481	0.95	12.84 ± 0.06	101.10	12.70 ± 0.06	100.00	25.41 ± 0.05	100.04

Table S2. Carbon fiber length in vPA12/CF and rPA12/CF composite filament

Statistical parameter	vPA12/CF	rPA12/CF
L_{min}, Minimum CF length (mm)	0.030	0.040
Q_1 (25th Percentile) (mm)	0.120	0.080
Q_2 (50th Percentile) (mm)	0.190	0.140
Q_3 (75th Percentile) (mm)	0.263	0.188
IQR (Interquartile Range) (mm)	0.143	0.108
L_{max}, Maximum CF length (mm)	0.510	0.430
Number-Average (Total arithmetical mean) (mm)	0.204	0.147
Weight-Average (mm)	0.254	0.191
Standard Deviation (mm)	0.106	0.076

Table S3. Summary of the ANOVA of the tensile tests (dog-bone samples)

Dog-bone samples	Young's modulus (MPa)		Tensile strength (MPa)		Stress at break (MPa)		Strain at break (-)	
**Source**	**P-Value**	**C (%)**	**P-Value**	**C (%)**	**P-Value**	**C (%)**	**P-Value**	**C (%)**
**Recycling (-)**	0.014	3.45	0.091	1.15	0.173	10.59	0.017	69.81
**Orientation (°)**	0.001	96.46	0.002	98.61	0.055	84.52	0.078	27.83
**Error**		0.10		0.24		4.89		2.36
	R^2 = 99.90%		R^2 = 99.76%		R^2 = 95.12%		R^2 = 97.63%	

Table S4. Summary of the ANOVA of the tensile tests (strip samples)

Tensile strip samples	Young's modulus (MPa)		Tensile strength (MPa)		Stress at break (MPa)		Strain at break (-)	
**Source**	**P-Value**	**C (%)**	**P-Value**	**C (%)**	**P-Value**	**C (%)**	**P-Value**	**C (%)**
**Recycling (-)**	0.032	7.27	0.045	20.37	0.861	1.25	0.751	3.18
**Orientation (°)**	0.005	92.24	0.024	77.69	0.640	35.60	0.496	48.76
**Error**		0.47		1.94		63.15		48.04
	R^2 = 99.50%		R^2 = 98.06%		R^2 = 36.85%		R^2 = 51.95%	

Table S5. Summary of the ANOVA of the three-point bending tests

Three-point bending samples	Bending modulus (MPa)		Bending strength (MPa)	
**Source**	**P-Value**	**C (%)**	**P-Value**	**C (%)**
**Recycling (-)**	0.057	5.34	0.089	17.62
**Orientation (°)**	0.007	94.00	0.044	78.79
**Error**		0.66		3.59
	R^2 = 99.34%		R^2 = 96.41%	

Table S6. Summary of the ANOVA of the compression tests

Compression samples	Compression modulus (MPa)		Compression strength (MPa)	
**Source**	**P-Value**	**C (%)**	**P-Value**	**C (%)**
**Recycling (-)**	0.02	25.40	0.065	13.76
**Infill pattern (-)**	0.002	62.98	0.014	68.81
**Flow (%)**	0.066	5.30	0.553	1.08
**Error**		6.32		16.36
	R^2 = 93.68%		R^2 = 83.64%	
